# Sensitive detection of structural dynamics using a statistical framework for comparative crystallography

**DOI:** 10.1126/sciadv.adj2921

**Published:** 2025-12-03

**Authors:** Doeke R. Hekstra, Harrison K. Wang, Margaret A. Klureza, Jack B. Greisman, Kevin M. Dalton

**Affiliations:** ^1^ Department of Molecular and Cellular Biology, Harvard University, Cambridge, MA 02138, USA.; ^2^ School of Engineering and Applied Sciences, Harvard University, Boston, MA 02134, USA.; ^3^ Graduate Program in Biophysics, Harvard University, Boston, MA 02115, USA.; ^4^ Department of Chemistry and Chemical Biology, Harvard University, Cambridge, MA 02138, USA.; ^5^ Linac Coherent Light Source, SLAC National Accelerator Laboratory, Menlo Park, CA 94025, USA.; ^6^ Department of Biology, New York University, New York, NY 10003, USA.

## Abstract

Chemical and conformational changes are crucial to protein function and its pharmacological control. X-ray crystallography can reveal these changes in atomic detail, but standard analysis methods, which refine separate datasets, often overlook differences that are subtle or arise in only a subset of molecules. Direct comparison of crystallographic datasets is, in principle, more powerful, but systematic errors (“scales”) often mask changes in the crystallographic observables (“structure factors”). Machine learning algorithms that jointly estimate scales and structure factors can address this limitation. Here, we augment this approach with multivariate, structured priors derived from crystallographic theory, implemented in the variational deep learning framework Careless. Doing so strongly improves the detection of protein dynamics, element-specific anomalous signals, and the binding of drug candidates, offering a robust approach to comparative crystallography and, potentially, to detection of protein dynamics by other structure determination methods.

## Teaser

Statistical modeling of similarity between crystallographic datasets strongly improves detection of structural dynamics.

## INTRODUCTION

**Fig. 1.**
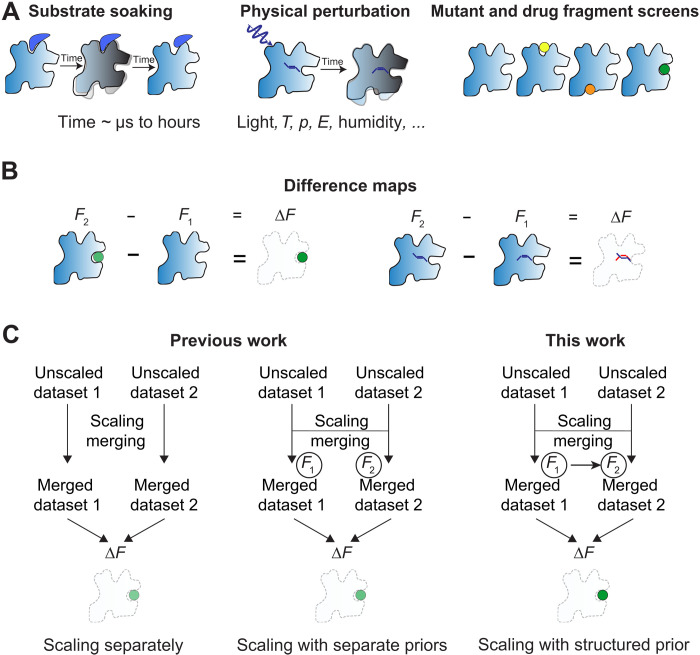
Schematic overview of comparative crystallography and this work. (**A**) Schematic overview of comparative crystallography experiments. (**B**) Subtracting structure factor amplitudes (top) from isomorphous datasets results in spatial “difference maps” displaying structural differences as changes in electron density. (**C**) Schematic overview of scaling comparative crystallography data. Previous work involved scaling datasets separately (left) or jointly with separate priors (center), while this work involved scaling datasets jointly and with a structured prior (right), which can produce stronger signal in difference maps.

Proteins and their assemblies are dynamic molecular machines that mediate chemical catalysis, molecular transport, signal transduction, and the allosteric control of these processes. To understand the function of these machines, one typically needs to monitor their functional and structural response to perturbations. We illustrate this paradigm with several examples in Fig. 1A. For example, foundational to our understanding of DNA polymerases, the motion of key residues and the substrate of DNA polymerase were revealed by soaking magnesium into crystals of a DNA polymerase (*1*–*3*) with observation by x-ray crystallography. Physical perturbations (e.g., light, temperature jump, and electric field) have also been used to activate the functional dynamics of proteins involved in signal transduction (*4*, *5*), oxygen transport (*6*–*8*), ion transport across membranes (*9*–*11*), and DNA repair (*12*) in crystallographic experiments visualizing key interactions and intermediates with high spatial and temporal resolution. Last, the discovery of drug precursors targeting key enzymes involved in severe acute respiratory syndrome coronavirus 2 (SARS-CoV-2) replication (*13*–*15*) and pathological signal transduction (*16*) has been addressed by adding libraries of thousands of potential ligands to protein crystals. In addition to identifying medically relevant compounds, these ligand-screening efforts contribute insight into the fundamental biophysical mechanisms of proteins (*17*).

X-ray crystallography makes it possible to measure the effect of these perturbations with high spatial and temporal resolution, over a wide range of timescales, and with high throughput. Recent methodological advances have expanded this capability markedly, because of new x-ray sources such as fourth-generation synchrotrons (*18*) and x-ray free-electron lasers (XFELs) (*19*, *20*), new time-resolved methods such as mix-and-inject studies of enzyme catalysis (*21*–*25*), and high-throughput methods permitting screening of thousands of molecules’ interactions with proteins of interest (*13*–*16*, *26*, *27*). High-resolution data collected from these methods can enable the tracking of enzyme catalysis in atomic detail, mapping of protein dynamics (*16*, *28*–*30*), and the identification of drug building blocks (“fragments”) binding to their targets. These experiments provide useful insights for enzyme engineering and drug design and complement methods such as cryo–electron microscopy (cryo-EM) and solution scattering. Prospectively, structural data provided by these comparative crystallographic experiments also provide valuable training data for generative models of protein dynamics (*31*). Although crystal lattice contacts can affect large-scale motions, the weak interactions shaping most protein crystals often allow enzymes to remain active in crystalline form (*1*, *3*, *32*–*39*) and functional dynamics to be triggered by light or rapid mixing with substrate, enabling direct observation by time-resolved x-ray crystallography of biological processes out of equilibrium (*4*, *6*, *9*, *10*, *12*, *21*, *40*–*47*). These observations complement observations of equilibrium dynamics by nuclear magnetic resonance (NMR) spectroscopy as well as biochemical and cell-based functional assays.

**Fig. 2.**
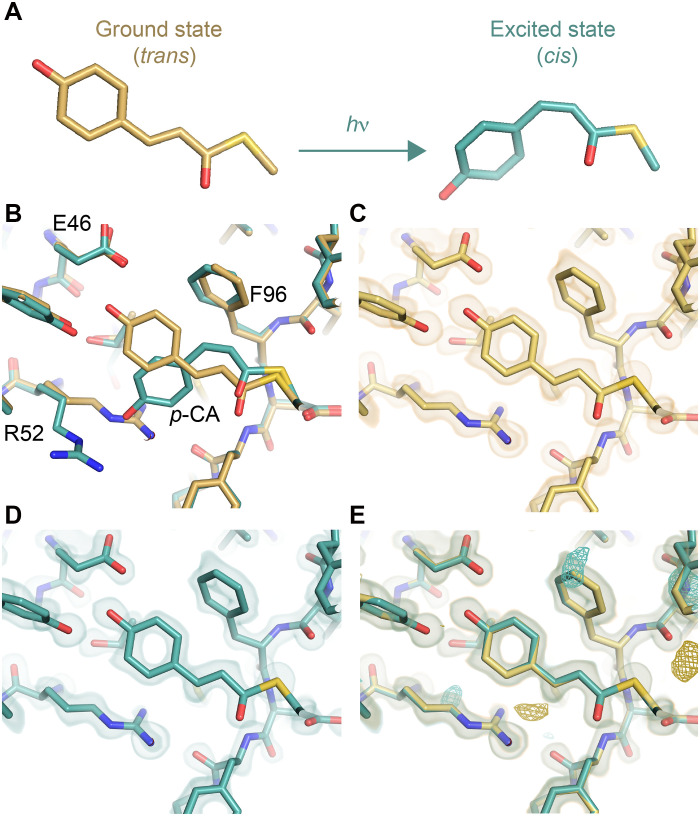
Schematic overview of comparative crystallography and this work. (**A**) The *p*-coumaric acid (*p*-CA) chromophore of PYP isomerizes when exposed to blue light [adapted from previous work (*54*)]. (**B**) Structural context of the PYP chromophore from Protein Data Bank (PDB) ID 1TS0, in both conformations, with nearby residues indicated. (**C** and **D**) Model of PYP refined against the dark (C) and 2-ms (D) observations before and after exposure to blue light, respectively. The electron density map in each panel is the 2*mF*_o_ − *DF*_c_ map from merging with Careless and refining starting from the ground-state model PDB ID 2PHY. These maps have been contoured to 2σ and carved to 1.5 Å of the model. (**E**) Overlay of models and maps from (C) and (D), along with the *F*_o_ − *F*_c_ electron density of the 2-ms map and model, a teal and yellow mesh contoured to +3σ and −3σ, respectively.

Often, conformational changes in a protein are below the detection threshold of conventional analysis methods. We consider a paradigmatic example: two models of photoactive yellow protein (PYP) refined against time-resolved crystallography datasets taken before and 2 ms after exposing the crystal to blue light (henceforth dark and 2-ms datasets, respectively). The light-induced *trans*-to-*cis* isomerization of the active site chromophore (Fig. 2, A and B) in PYP is a conformational change that has been studied extensively (*48*–*50*). Refinement of single models against each of the dark and 2-ms datasets fails to detect this conformational change, revealing no structural differences at all (Fig. 2, C and D). This conclusion is underscored by the *F*_o_ − *F*_c_ map, which crystallographers use to assess the agreement between a structure and the experimental data. No difference density appears in the vicinity of the chromophore, indicating that the model appears to agree with the data within experimental error (Fig. 2E and fig. S1). In the end, to resolve the isomerization of the chromophore, the original study did not compare refined structures but visualized changes in electron density using an isomorphous difference map (Fig. 1B) and then refined against differences between the datasets, in a method called extrapolated structure factor (ESF) refinement (*51*, *52*). ESF refinement is popular for inferring perturbed state models in experiments where only a small portion of the crystal has been perturbed (*53*), as is typically the case in nonequilibrium time-resolved experiments and drug fragment screening wherein the fragments typically have low affinity for the protein target. Although ESF refinement has been successful, it relies on merged structure factors that can obscure upstream inaccuracies in data processing. We asked whether we could obtain better sensitivity by considering the comparative nature of crystallography earlier in data reduction.

Here, we introduce a framework that enables statistically efficient comparison of related datasets (Fig. 1C). This method operates at the time of “scaling,” during which systematic errors in the unmerged observations are estimated and corrected. We first observe that the structures of proteins at different time points or under different chemical or physical conditions are often very similar and therefore provide nearly redundant measurements. We then formalize this observation into a family of multivariate priors that accurately captures correlations between related crystallographic datasets and implement these structured priors in a software package, Careless, that can scale and merge diffraction data from all major experimental modalities at synchrotrons and XFELs (*54*), as well as microcrystal electron diffraction data (*55*). Careless is based on approximate Bayesian inference combined with a deep neural network that learns scale factors from experimental metadata. By comparing related datasets, we attain large improvements in the detection of signals for key applications—detection of protein dynamics from polychromatic, time-resolved experiments, detection of specific elements in enzymes from serial synchrotron and XFEL experiments, and the detection of bound drug precursors in a large drug fragment screen. In concurrent work (*43*, *56*), we further show that this approach enables direct observation of enzyme catalysis in full spatial and temporal detail. We anticipate that structured priors will broadly improve the detection of structural differences arising from general perturbations across a variety of comparative crystallography experiments.

## RESULTS

### A statistical framework for comparative crystallography

**Fig. 3.**
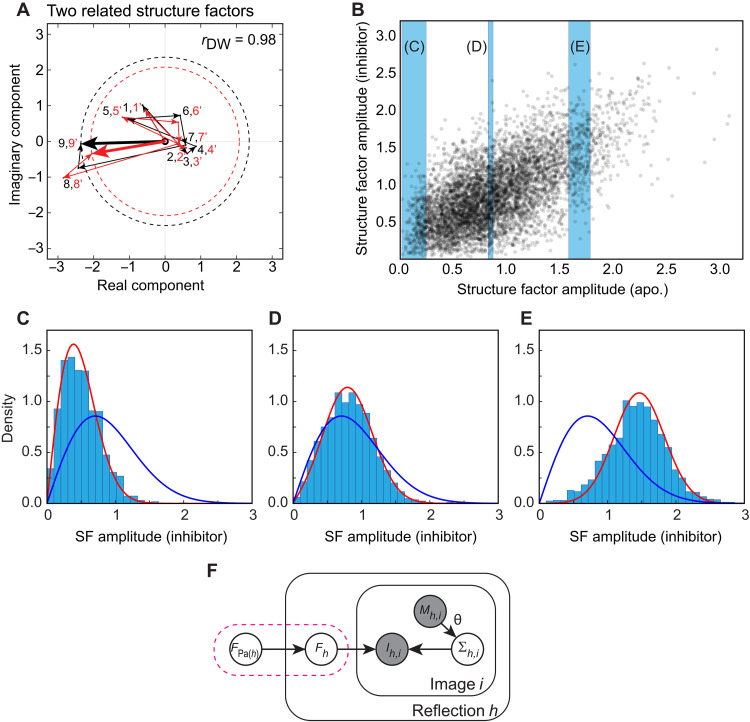
The double-Wilson model and its implementation in Careless. (**A**) A simulation of complex structure factors of related structures (thick black and red arrows), built up from nine pairs of contributions from individual atoms (numbered black and red arrows). In each atom pair, the contribution from each atom is random but correlated with correlation *r*_DW_ between the real components and likewise between the imaginary components. The experimentally observed amplitudes of the structure factors are described by the radii of the dashed circles. (**B**) Scatter plot for structure factor amplitudes for isomorphous PTP-1B datasets for apo. and inhibitor-bound forms (for a random subset of Miller indices). Blue slices indicate data points for which histograms are shown in (C) to (E). (**C** to **E**) Histograms for slices through (B) are better approximated by the Rice distributions (red) parametrized by a global *r*_DW_ (here, 0.85) than by the unconditional, univariate Wilson distribution (blue). (**F**) A graphical model illustrating the probabilistic model underlying Careless: Structure factor amplitudes *F_h_* and scaling corrections Σ*_h_* are inferred from observed diffraction intensities *I_h,i_* and reflection- and image-specific metadata *M_h,i_*. Pink dashed box indicates accounting for expected correlations with a similar structure [the “parent” Pa(*h*) of reflection *h*] improves inference.

We first briefly outline our framework for comparing crystallographic datasets, which is explained in mathematical detail in Supplementary Text. To begin, we note that the complex structure factors that comprise crystallographic datasets are the sum of contributions from each constituent atom, demonstrated as a nine-atom simulation in Fig. 3A. In this situation where the statistical observable is a sum of many random variables, it is reasonable to invoke the central limit theorem, which states that the sum of these variables will follow a normal distribution. Here, each pair of structure factors will follow a bivariate normal distribution in the complex plane. The structure factor amplitudes, which correspond to the distance from the origin in Fig. 3A (dashed circle), follow the well-known Wilson distribution (*57*). Small changes in the positions of constituent atoms, resulting from a perturbation, result in a small change in the total structure factor. In Supplementary Text, we show that two related datasets will closely follow a multivariate normal distribution characterized by a correlation parameter, henceforth the double-Wilson *r* (*58*, *59*), that quantifies the correlation between the real components of the structure factors. We refer to this statistical model as the double-Wilson model.

To illustrate with a pair of related datasets, Fig. 3B compares structure factor amplitudes for crystals of human protein tyrosine phosphatase 1B (PTP-1B) in the presence and absence (apo.) of the inhibitor TCS-401 (*60*). A fair degree of correlation is evident. A fit to the double-Wilson model (see Supplementary text S2.3) shows that *r* ≈ 0.85, with the implied Pearson correlation coefficient between structure factor amplitudes ≈ *r*^2^ (Supplementary text S5) (*61*). Histograms of structure factor amplitudes for the inhibitor-bound data (Fig. 3, C to E, and fig. S2), given the binned amplitudes of the apo. data (blue shading in Fig. 3B), are better fit by this model (red curves in Fig. 3, C to E) than by assuming statistical independence (blue curves). We likewise find that the double-Wilson model describes other comparative crystallography datasets well, including time-resolved x-ray crystallography experiments (fig. S3), replicate datasets across laboratories (fig. S4), and across temperatures (fig. S5), providing confidence that the formalism generally applies to related datasets.

### Scaling of related datasets

Crystallographic datasets inevitably contain systematic errors resulting from numerous physical processes, which confounds detection of dataset differences. These systematic errors are challenging to fully remove (see Supplementary Text S1). We previously introduced a Bayesian statistical framework that simultaneously learns structure factor amplitudes *F_h_* and scales Σ*_h,i_* from the observed intensities *I_h,i_*. This framework includes variational approximations of the posterior distributions of the structure factor amplitudes and a multilayer perceptron (with parameters θ) to predict scales from reflection metadata (such as Miller indices, image number, position on the detector, and x-ray wavelength) (*54*). The basic inference challenge is illustrated in Fig. 3F using a probabilistic graphical model (*62*). As in most conventional approaches, we had treated related structure factor amplitudes as statistically independent. However, for comparative applications, we hypothesize that we can better constrain inference using the additional “pseudoredundancy” afforded by simultaneous inference of similar structure factor amplitudes, which could strongly constrain inference [shown in the pink box in Fig. 3F as conditioning of *F_h_* on a “parent amplitude” *F*_Pa(*h*)_]. To test this idea, we implemented the multivariate Wilson distribution as a prior in Careless. We provide guidelines for use of Careless in Supplementary Text and in (*56*, *63*).

### Laue anomalous diffraction

Although many x-ray sources are intrinsically polychromatic, most of their photons are typically discarded by use of monochromators to simplify data analysis. For studies of dynamics, however, it can be essential to use the much brighter polychromatic pulses, giving rise to Laue diffraction. To assess whether the use of multivariate priors can improve the processing of Laue data, we first addressed the extraction of anomalous signal, which can be recovered from small differences between Bijvoet pairs—pairs of structure factors that are identical in the absence of element-specific electronic resonances (i.e., anomalous effects) but that differ when these effects are present. Observation of anomalous signal on specific atoms depends critically on stringent removal of systematic errors.

**Fig. 4.**
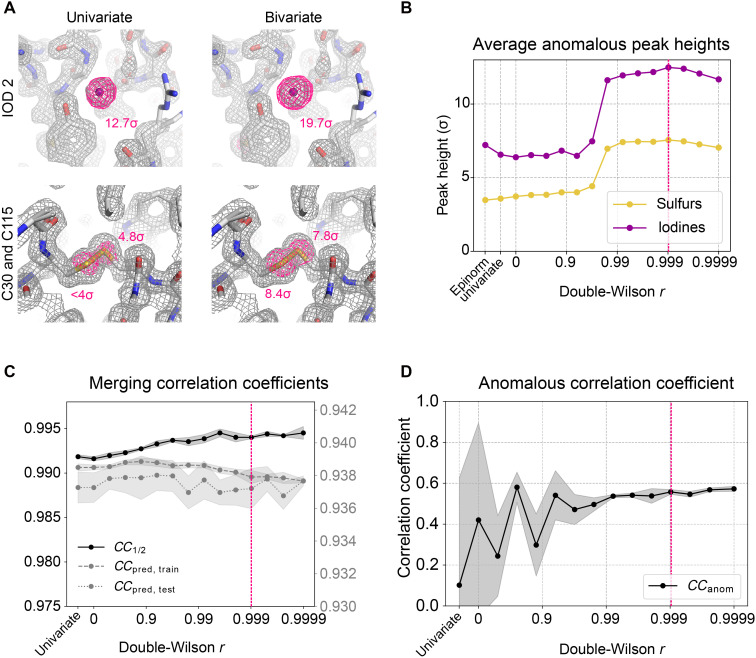
Use of a bivariate prior improves anomalous signal in a Laue diffraction experiment. (**A**) Comparisons between anomalous omit peaks after scaling and merging with univariate or bivariate priors [using *r* = 0.999 for the bivariate prior, marked in (B) to (D) with a dashed magenta line]. The observed electron density map (2*mF*_o_ − *DF*_c_) in gray is contoured at 1.5σ, and the anomalous difference omit map in magenta is contoured at 6σ for iodine (IOD) 2 and 4σ for C30 and C115. (**B**) Dependence of average iodine and sulfur anomalous peak height on *r*. Additional peak heights are plotted for merging with the univariate prior or with Epinorm. (**C**) Merging correlation coefficients of the lysozyme dataset across double-Wilson *r* values. The *y*-axis labels for the *CC*_1/2_ are on the left, and the *y*-axis labels for the *CC*_pred_ are on the right. A test set of 10% of observations were held out during scaling and merging to evaluate performance of the scaling model, yielding *CC*_pred, test_ for the test set and *CC*_pred, train_ for the 90% of data used during scaling. The shaded confidence interval of the *CC*_1/2_ curve represents the SD over three random half-dataset repeats, and the shaded confidence interval for the *CC*_pred_ curves represent the SD over three Careless runs. (**D**) Anomalous correlation coefficient, *CC*_pred_, of the lysozyme dataset across *r*. The shaded confidence interval represents the SD over three random half-dataset partitions.

In this example, the data were collected on a single crystal of hen egg white lysozyme soaked with sodium iodide using polychromatic x-ray pulses (about 5% spectral bandwidth, with wavelength from 1.02 to 1.20 Å). As these data were obtained from a single crystal, one might expect little inconsistency across the data and minimal benefit from imposing a bivariate prior on Bijvoet pairs. Nevertheless, we compared scaling and merging in Careless with a univariate Bayesian prior (i.e., the standard Wilson distribution) against a bivariate prior that assumes varying levels of correlation, *r*, between the Bijvoet mates. For consistency, we phased all sets of structure factor amplitudes using a reference model refined against monochromatic data collected on the same day. In the resulting anomalous difference map, we find strong gains in peak heights of both iodide ions and sulfur atoms when using a bivariate prior (Fig. 4A and Fig. S6A), although the anomalous scattering strength of sulfur is equivalent to only about one-fourth of an electron at the dominant wavelength of the incident x-ray spectrum (*f″* = 0.26 at 1.04 Å). The signal strength is maximal at an intermediate *r* of 0.999, with an increase in anomalous peak height of about 70% over the use of a univariate prior (Fig. 4B and fig. S6B). As a guide to choosing *r*, we recommend scanning a range of possible values. A good estimate can typically be obtained within minutes by scaling and merging a small batch of data (see fig. S7, where we processed the first 12.5% of data). Alternatively, Careless also supports automatic optimization of *r* (see Supplementary Text).

Cross-validation measures of data processing quality—*CC*_1/2_ (*64*), *CC*_pred_ (*63*), and *CC*_anom_ (*65*)—are also improved by the introduction of the bivariate prior (Fig. 4, C and D). Careless with a bivariate prior also strongly improves over the long-standing state of the art for scaling polychromatic diffraction data, Epinorm (Renz Research Inc.), increasing anomalous signal by 73% for iodine atoms and 118% for sulfur atoms. Notably, the anomalous signal increases substantially after the double-Wilson *r* exceeds about 0.96 (Fig. 4B). The same trend was observed when the images were processed with the open-source package Laue-DIALS (*66*) (figs. S8 and S9).

### Time-resolved Laue diffraction

**Fig. 5.**
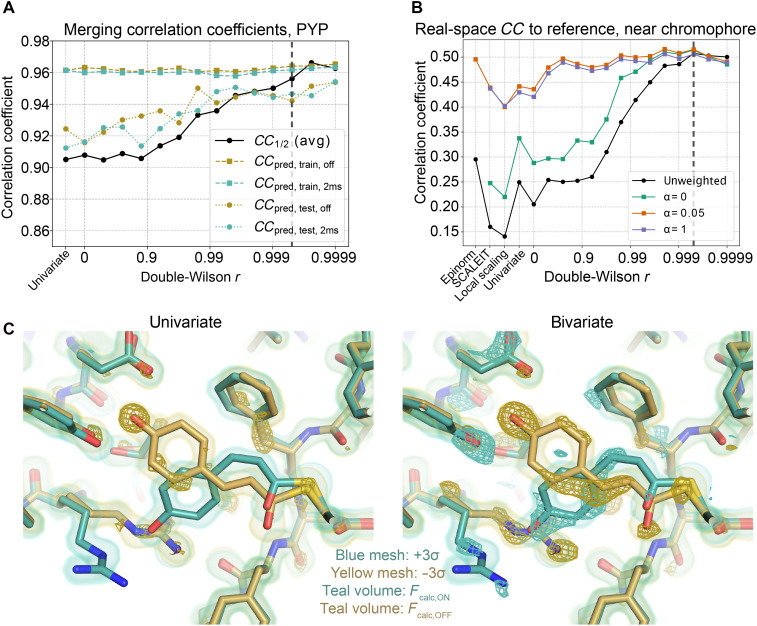
Use of a bivariate prior improves time-resolved difference signal. (**A**) Merging statistics of the 2-ms and dark structure factor amplitudes as a function of *r*. (**B**) Correlation coefficient of the weighted (α = 0, green; α = 0.05, red; α = 1, blue) and unweighted (black) observed difference maps with the 2 ms − dark calculated difference map (*F*_calc,2ms_ − *F*_calc,OFF_), in a region 10 Å around the chromophore. α weights the contributions of large structure factor differences (see Materials and Methods). The correlation coefficient is plotted as a function of the double-Wilson parameter *r*. Benchmarks included are as follows: (i) Epinorm, (ii) Careless with univariate prior, and Careless with the univariate prior followed by (iii) local scaling (*103*) or (iv) SCALEIT (*104*). Vertical dashed lines in (A) and (B) indicate the *r* value, 0.9995, that parametrizes the bivariate prior for merged data displayed in (C). (**C**) Left: Observed difference map (teal and yellow mesh, contoured to 3σ, unweighted), from merging with a univariate prior. Τhe map is overlaid with models (2-ms and dark models colored with teal and yellow sticks, respectively) and the electron density at 2 ms after excitation and in the dark state (teal and yellow surfaces, respectively; contoured to 1.5σ), based on calculated structure factors *F*_2ms_ and *F*_dark_ from PDB entry 1TSO. Right: Isomorphous 2-ms − dark difference map (yellow and teal mesh, contoured to 3σ, unweighted), from merging with a bivariate prior, *r* = 0.9995. *F*_2ms_ map and *F*_dark_ map are shown in the left panel.

Laue diffraction at synchrotron sources provides access to the dynamics of proteins on timescales of 100 ps and longer. To determine whether a bivariate prior can improve scaling of time-resolved signal, we processed a time-resolved Laue dataset collected on a single crystal of PYP at BioCARS 14-ID at the Advanced Photon Source. Our dataset, the same as in Fig. 1, contains 20 images of PYP without laser exposure and 20 images 2 ms after exposure to a blue laser pulse, at which point PYP is expected to adopt an intermediate state (*50*). Using Careless, we jointly scaled the dark and 2-ms datasets while imposing a bivariate prior with varying levels of the correlation parameter, *r*. Merging statistics improve when scaling with a bivariate prior (Fig. 5A).

The extensive literature on PYP enables us to quantify the quality of the resulting electron density difference maps: On the basis of models of the ground-state and excited-state conformations from previous studies, we can calculate correlation coefficients between observed and predicted difference maps. At the best value of *r* (about 0.9995), this real-space correlation coefficient is more than 0.50 within 10 Å of the chromophore. Without weighting (*67*), this correlation coefficient is about twice as strong as for an observed difference map obtained by either scaling with a univariate prior or scaling with Epinorm, the state of the art for scaling Laue diffraction data (Fig. 4B). Consistent with this quantitative assessment, the difference map near the chromophore is more readily visually interpretable after scaling with a bivariate prior (Fig. 5C and fig. S10) than without. A scan of difference map quality on a small subset of the data again suffices to select *r* (fig. S9A). We note that alternative approaches that adjust scales after merging can sometimes improve visual interpretability (fig. S11) but are not, in this case, effective in improving correlation with the calculated maps (“SCALEIT” and “local scaling”; Fig. 5B and see Supplementary Text for further discussion). That is, our approach improves detection of dynamics from time-resolved x-ray data over state-of-the-art methods.

Last, it is instructive to compare our scaling approach with commonly used weights (*8*, *29*, *53*, *54*) that are heuristic modifications of a weighting scheme proposed in (*67*), inspired by Bayesian statistics. These weights reduce the contributions of estimated structure factor amplitude differences (Δ*F*) that are large or have large estimated errors [σ(Δ*F*)]. A coefficient α, used in calculating the weights, tunes the contribution of large Δ*F* to the difference map (see Materials and Methods). In the case of the PYP data (Fig. 5C), we find that suppression of large |Δ*F*| (by choosing α > 0) is necessary to achieve a similar effect as the bivariate prior. That is, the bivariate prior suppresses spuriously large contributions to the difference map without the need for heuristic weighting schemes.

### Serial femtosecond crystallography

**Fig. 6.**
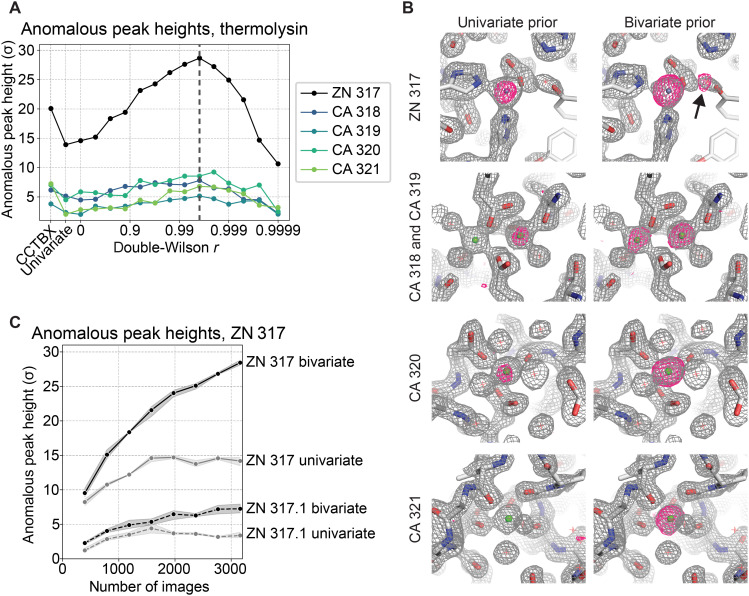
Scaling and merging with a bivariate prior improves the anomalous signal in a serial XFEL experiment. (**A**) Anomalous difference map peak heights in thermolysin, shown for zinc and four calcium sites, as a function of the double-Wilson parameter *r*. Additional peak heights are shown for merging with the univariate Bayesian prior and merging with CCTBX (*54*, *70*). Vertical dashed black line in (A) indicates the *r* value, 0.9961, that parametrizes the bivariate prior for merged data displayed in the following panel. (**B**) First row: Region near the thermolysin-bound zinc atom, with observed electron density map (2*mF*_o_ − D*F*_c_) in gray, contoured at 2σ, and anomalous difference omit map in magenta, contoured at 5σ. Arrow indicates the location of an alternative zinc binding site revealed by merging with the bivariate prior. Next three rows: Anomalous difference omit maps of thermolysin calcium sites, carved to 1.5 Å of the model and contoured to 4σ (magenta). (**C**) The anomalous peak height of ZN 317 and its alternate site, ZN 317.1, as a function of the number of frames, at *r* = 0.9951. Shaded band represents 95% confidence interval over three independent scaling repeats.

Serial femtosecond x-ray crystallography (SFX) of microcrystals probed by intense XFEL pulses has markedly expanded the range of systems and timescales accessible to time-resolved studies. To determine whether multivariate priors could also improve the accuracy of SFX data processing, we sought to extract anomalous signal from an SFX dataset collected for thermolysin, an enzyme containing a catalytic zinc (Zn) ion in its active site. Specifically, we processed 3160 images from a much larger dataset [Coherent X-ray Imaging Data Bank (CXIDB) 81] (*68*, *69*). To account for the serial monochromatic data collection strategy, we included an Ewald offset and per-image layers in our scaling model, as before (*54*). We split Bijvoet pairs into two half datasets before relating these half datasets by a joint prior during scaling in Careless. The anomalous difference map peak height of the catalytic zinc ion (ZN 317) increases from 15σ without a bivariate prior to 28σ for the optimal *r* value, surpassing state-of-the-art XFEL-specific software [Computational Crystallography Toolbox (CCTBX); Fig. 6A] (*54*, *70*). We also find that two estimation methods can get us close to the best *r* value found by our scan: use of a small subset of data (fig. S12A) or direct optimization of *r* in Careless (fig. S13).

As *r* is varied, we can detect more anomalous sites. First, more associated calcium ions become detectable. Using a univariate prior, only two of four calcium ions appear above 3σ (an approximate noise threshold). After scaling with a multivariate prior, all four calcium ions are detectable (Fig. 6, A and B). Second, in prior analyses of a thermolysin dataset with four times as many diffraction images, anomalous difference signal attributable to a low-occupancy zinc ion was detected at a site adjacent to catalytic zinc ion ZN 317 (*68*, *71*). When we previously scaled our smaller dataset with a univariate prior, this site (ZN 317.1) was not detectable (*54*). Upon scaling with a multivariate prior, however, this site can be clearly identified (Fig. 6B, black arrow), emerging above the noise with as few as ~1000 diffraction images (Fig. 6C). We note that these anomalous difference maps can also be improved by weighting, but this is less effective than the use of a multivariate prior during scaling (fig. S12C). In summary, a bivariate prior strongly improves the detection of anomalous signal from SFX data.

### Fragment screening

Last, crystallographic drug fragment screens now enable the high-throughput discovery of pharmacological lead compounds against proteins of interest, e.g., SARS-CoV-2 proteins (*13*–*15*, *26*). In these studies, thousands of crystals of a protein of interest are each soaked with a “drug fragment”—a small-molecule building block for larger drug molecules. To determine whether joint scaling can improve the detection of bound fragments, we analyzed data from a fragment screening experiment totaling 1 apo. dataset and 16 holo datasets identified by PanDDA (Pan-Dataset Density Analysis) (*72*), a state-of-the-art algorithm based on real-space electron density map manipulations. This dataset contains structures of SARS-CoV-2 nonstructural protein 3 macromolecular domain 1 (Nsp3 Mac1) with ligands from a fragment screening library bound to the Mac1 adenosine-binding site.

**Fig. 7.**
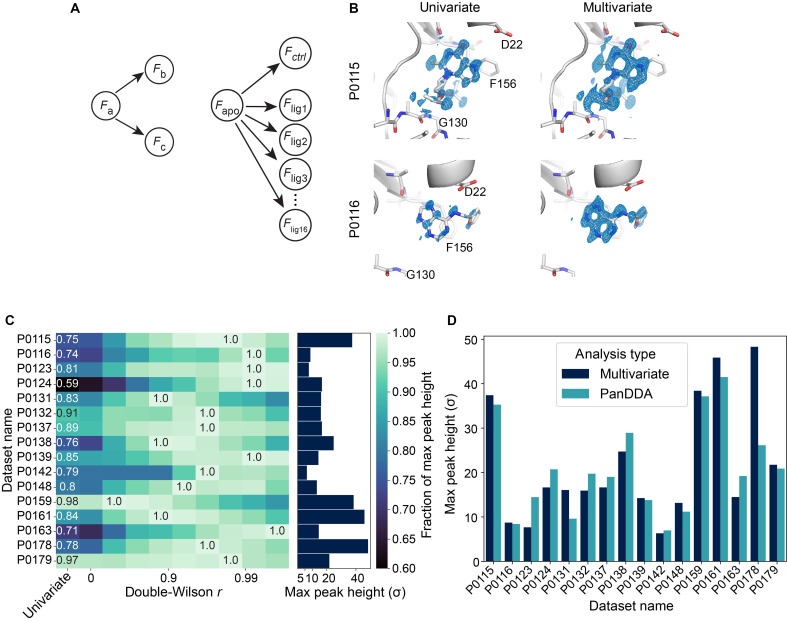
Use of a multivariate prior improves bound fragment signal in a drug fragment screen. (**A**) A graphical model (or Bayesian network) illustrating the relationship between three datasets (or, more generally, sets of structure factor amplitudes). Arrows indicate conditional dependence; the lack of an arrow between datasets *F*_b_ and *F*_c_ indicates conditional independence. (**B**) holo − apo. isomorphous difference maps from fragment screening of Mac1, scaled with a univariate or multivariate prior. All maps carved to 1.5 Å from the ligand and contoured to +3σ. (**C**) Tallest positive peak in each holo − apo. difference map as a function of the double-Wilson *r* parameter. For a given holo dataset, the tallest positive peak is measured. The maximum peak height of that peak, across all double-Wilson *r* values, is normalized to 1.0. The maximum peak height is labeled and reported in a bar chart (right). Also labeled is the fraction of the maximum peak height for the tallest peak in the univariate datasets (left). The *r* value, for each dataset’s maximum peak height, parametrizes the bivariate prior for merging the electron density displayed in (B). (**D**) Comparison of each dataset’s ligand peak heights as processed with PanDDA or Careless with bivariate prior.

The statistical model generalizes to more than two datasets. In this case, each dataset is represented by a node in a Bayesian network or graphical model (Fig. 7A, left) (*62*). Edges (arrows) represent conditional dependence between datasets. Whenever this graph is acyclic, the resulting joint probability of structure factor amplitudes can be calculated analytically (see Supplementary Text). We will refer to the resulting probability distribution as the multivariate Wilson distribution. The expected correlations in many comparative crystallography experiments can be modeled by these networks (Fig. 7A, right, and fig. S14). Our implementation of the multivariate Wilson model expects users to supply the topology of conditional dependencies between datasets and an estimate of the double-Wilson parameter *r*. For practical guidelines on this approach, see Materials and Methods, Supplementary Text, and (*56*).

Following this paradigm, we ran Careless on unmerged intensities, adding several metadata keys, including one-hot encodings of each dataset (see Materials and Methods). By one-hot encoding, we allow the model to express differences in scale between fragment-bound datasets. In addition, we model each holo dataset as dependent on the apo. dataset, while assuming conditional independence between holo datasets (Fig. 7A, right). We find that difference density maps visually improve upon scaling with a multivariate prior (Fig. 7B and fig. S14). We tracked the largest ligand peaks in the difference map for a given dataset, while we varied *r*. We find that in all datasets, this signal is strongest when scaling with a multivariate prior (Fig. 7C). The *r* value for the tallest peaks differs between holo datasets. Without special modifications to scaling and merging, we obtain peak heights on par with those produced by PanDDA (Fig. 7D and table S2). We note that PanDDA analysis outperforms difference map analysis after scaling with a bivariate prior when measured by real-space correlation with a reference difference map (fig. S16A) calculated from bound and apo. models generated by PanDDA. This real-space correlation diminishes more strongly for PanDDA when the ligand mask is expanded (fig. S16B). This may be because PanDDA warps electron density maps using a structure-guided map alignment algorithm, which may suppress the detection of protein conformational changes. Since PanDDA analysis is performed after scaling and merging, further gains may be possible by combining our scaling method with PanDDA.

To determine whether the correlation between apo. and holo datasets, measured by r, relates to ligand occupancy, we examined the relationship between optimal *r* and the PanDDA background density correction factor (BDC; a parameter that scales inversely with ligand occupancy and accounts for crystal-to-crystal variation in soaking and data quality) (*27*). We find a moderate correlation with double-Wilson *r* (Pearson *r* = 0.505, two-tailed *t* test: *P* < 0.05, *N* = 16) (fig. S16D). Merging statistics also improve when scaling with a multivariate prior (fig. S16E): in particular, the gap between the training and test *CC*_pred_ is reduced for higher *r*, indicating that the multivariate prior reduces overfitting of the scales.

## DISCUSSION

These results establish that a Bayesian formalism using multivariate priors enables machine learning methods to sensitively detect protein dynamics, chemical signals, and ligand binding from comparative crystallographic data by exploiting correlations among related datasets. In concurrent work, we show that the formalism also strongly aids identification of intermediate states of an enzyme during catalysis (*43*). Naturally, in each case, the biological significance of observed motions is best assessed in combination with other types of data, such as functional readouts of mutations suggested by the crystallographic observations. An intense focus by the community on the prediction and observation of protein dynamics, combined with rapid advances in XFEL and synchrotron instrumentation, suggests that our work will find wide application in protein engineering, drug discovery, and the study of protein dynamics.

We provide further use examples of Careless in its online documentation (*73*), explain use of the available options in (*63*), and describe an ablation study identifying factors affecting Careless’ performance in (*56*). We discuss best practices for use of the multivariate prior in Supplementary Text and in extended online descriptions for the cases presented here (*74*). The statistical framework introduced here is not specific to Careless and can be implemented in other software. We encourage adoption of this framework by other crystallographic data processing software. Furthermore, the current memory limitations of Careless will be addressed in a forthcoming software package (*75*) that also builds on the presented statistical framework.

Our formalism has been inspired by successful Bayesian approaches to the crystallographic phase problem (*59*) and refinement (*76*). Although the developed formalism proves very effective in the form presented here, we note several promising extensions. First, high-quality reference datasets are commonly measured for serial time-resolved crystallography experiments, but these data are not commonly used to improve scaling. These datasets can be added to the Bayesian networks shown in fig. S14 to better condition scaling. Second, we currently treat the correlation parameter r as a global hyperparameter, yet correlations may vary between pairs of datasets (e.g., as a function of ligand occupancy) and vary with resolution. These correlations could be estimated from the data. Third, as not every node in a Bayesian network needs to be observed, our approach allows for interpolation of missing observations, enabling alternative data collection strategies that trade temporal coverage and completeness per time point (*77*). Fourth, the developed framework implies a joint distribution of the unobserved crystallographic phases across related datasets (see Supplementary Text and fig. S17). Future algorithms for model building and structure refinement algorithms could take advantage of such information. Last, cryo-EM and NMR can also measure heterogeneity in samples. These approaches are currently low throughput. Cryo-EM is still limited to large domain motions at slower timescales, while NMR is difficult to apply to large proteins or complexes. Future advances in cryo-EM and NMR could enable similar comparative techniques to be developed, growing the scope of perturbative methods to study protein dynamics. Specifically, the presented formalism may be readily extended to other methods for which the Wilson distribution provides a natural prior such as single-particle cryo-EM (*78*, *79*) and coherent x-ray imaging (*80*), as well as measurements of disordered crystals (*81*).

To take a broader perspective, our framework demonstrates that comparing dataset differences is superior to comparing individually processed structures when it comes to detecting subtle differences. Small structural changes are fundamental to our understanding of key biophysical phenomena such as drug binding, structural dynamics, and the effects of mutations. However, the status quo for structural comparison is to refine individual models against individual datasets and then compare models. In this status quo paradigm, subtle differences between conformations are readily obscured by inconsistent corrections for artifacts. We advocate for using structured priors as early as possible in the analysis pipeline. We expect this conclusion to be applicable in many domains within structural biology and beyond.

## MATERIALS AND METHODS

### Implementation details

The multivariate Wilson distribution we present here is implemented as a prior distribution in the Careless software package. Careless uses gradient-based optimization to maximize a Bayesian objective function (*82*, *83*), which is the sum of the expected log likelihood of the observed data plus the Kullback-Leibler divergence between the structure factors and the prior distribution. The likelihood of the data describes the probability of the observed diffraction intensities, *I_h,i_*, conditional on scale Σ*_h,i_*, and structure factor amplitudes *F_h_*. The likelihood also accounts for the estimated errors $\sigma_{I_{h,i}}$ in the observed intensities. The objective function is computed as described in equations 21 and 22 of Dalton *et al.* (*54*) using reparametrized samples (*84*) from a truncated normal surrogate posterior (variational distribution). For clarity, we reproduce this objective function

$$\begin{array}{c} \text{ELBO}\left(\mu_{\mathrm{qF}},\sigma_{\mathrm{qF}},\theta \right)\\ \approx \sum_{s=1}^{S}\left[\sum_{i}\text{log }p\left(I_{h,i}|F_{h,s}^{2}\Sigma_{h,i,s},\sigma_{I_{h,i}}\right)-\sum_{h}\left(\text{log }q_{F_{h}}\left(F_{h,s}\right)-\text{log }p\left(F_{h,s}\right)\right)\right] \end{array}$$

The evidence lower bound (ELBO) depends on the parameters of the surrogate posterior distributions, *q_F_* and *q*_Σ_. *q*_Σ_ is parameterized by a neural network with parameters θ, whose architecture is described in previous work (*54*) *q_F_* is parameterized by independent truncated normal distributions for each reflection with location and scale parameters $\left\{\mu_{q_{F_{h}}},\sigma_{q_{F_{h}}}\right\}$ supported on [0,∞) for centric reflections and (0,∞) for acentrics. *I_h,i_* refers to the intensity of a particular observation *i* for reflection *h*. *s* indexes reparameterized samples from the surrogate posteriors

$$\begin{array}{c} F_{h,s}\sim q_{F_{h}}\\ \Sigma_{h,i,s}\sim q_{\Sigma_{h,i}} \end{array}$$

Reparameterization is implemented in the TensorFlow Probability library (*85*). The prior distribution enters the objective function through the ultimate term, log*p*(*F_h,s_*). Previously (*54*), we exclusively used a univariate Wilson distribution as a prior for this term

$$p\left(F_{h,s}\right)=\left\{\begin{array}{ll} \sqrt{\frac{2}{{\pi \varepsilon }_{h}}}\text{exp}\left(-\frac{F_{h,s}^{2}}{2\varepsilon_{h}}\right) & h\;\mathrm{is}\;\mathrm{centric}\\ \;\;\frac{2}{\varepsilon }F_{h,s}\text{exp}\left(\frac{F_{h,s}^{2}}{\varepsilon_{h}}\right) & h\;\mathrm{is}\;\mathrm{acentric} \end{array}\right.$$

where ε*_h_* is the multiplicity of the reflection *h*, an integer value determined by the crystal’s symmetry. Note that because the scale function Σ*_h_* is parametrized separately within Careless, it does not appear in expressions for *p*(*F_h,s_*).

One of the features of variational inference is its flexibility. In this work, we leverage the inherent flexibility of reparameterization-based variational inference to extend the Careless package with a multivariate prior distribution. To do so, the Careless command line interface requires users to supply separate unmerged datasets for each node they wish to model (as in Fig. 7A and fig. S14). Careless asks the user to specify the parent of each node and the expected double-Wilson parameter r. Internally, this changes how the prior is calculated as follows

$$p\left(F_{h,s}\right)=\left\{\begin{array}{cc} \text{FoldedNormal}\left[F_{h,s}\left|rF_{\text{Pa}\left(h\right),s},\right.\sqrt{\varepsilon_{h}\left(1-r^{2}\right)}\right] & h\;\mathrm{is}\;\mathrm{centric}\\ \text{Rice}\left[F_{h,s}\left|rF_{\text{Pa}\left(h\right),s},\right.\sqrt{\frac{\varepsilon_{h}}{2}\left(1-r^{2}\right)}\right] & h\;\mathrm{is}\;\mathrm{acentric} \end{array}\right.$$

where Pa(*h*) denotes the parent reflection of *h*. Where a particular reflection has no parent because it is the root node, the prior defaults to the univariate Wilson model. When the parent observation is systematically absent (0) because of crystal symmetry but the child observation is not constrained to be 0, we use the above expression with *F*_Pa(*h*),*s*_ = 0. The folded normal distribution’s probability density function is

$$\begin{array}{c} \text{FoldedNormal}\left(x|\mu ,\sigma \right)\\ =\text{Normal}\left(x|\mu ,\sigma \right)+\text{Normal}\left(x|-\mu ,\sigma \right)\\ =\frac{1}{\sigma \sqrt{2\pi }}\left\{\text{exp}\left[-\frac{1}{2}{\left(\frac{x-\mu }{\sigma }\right)}^{2}\right]+\text{exp}\left[-\frac{1}{2}{\left(\frac{x+\mu }{\sigma }\right)}^{2}\right]\right\} \end{array}$$

while that of the Rice distribution is

$$\text{Rice}\left(x|v,\sigma \right)=\frac{x}{\sigma^{2}}\text{exp}\left[-\frac{\left(x^{2}+v^{2}\right)}{2\sigma^{2}}\right]I_{0}\left(\frac{\mathrm{xv}}{\sigma^{2}}\right)$$

where *I*_0_ denotes the modified Bessel function of the first kind with order zero. For numerical stability, both probability densities are computed directly in log space.

### Photoactive yellow protein

Collection of the Laue diffraction data for PYP was described previously (*86*). We performed indexing, geometry refinement, wavelength assignment, and integration using Precognition version 5.2.2 (Renz Research Inc.). Precognition-integrated intensities were written to mtz files and then scaled and merged with Careless version 0.4.1, with use of a bivariate prior on the dark and 2-ms time point mtz files. Fifteen different double-Wilson *r* values were used, with *r* varied for each Careless run as 1 to 0.5^run number^. Epinorm (Renz Research Inc., as bundled with Precognition) was also used for scaling and merging these data. Correlation coefficients were computed using careless.cchalf, careless.ccpred, and custom scripts based on GEMMI version 0.6.2 (*87*) and reciprocalspaceship version 1.0.1 (*88*). Isomorphous difference maps were first phased with a dark model refined in PHENIX version 1.20.1 (*89*) from Protein Data Bank (PDB) ID 2PHY (*90*) and then weighted using reciprocalspaceship according to

$$w_{\mathrm{hkl}}={\left(1+\frac{\sigma^{2}\left(∆F\right)}{\langle \sigma^{2}\left(∆F\right)\rangle }+\alpha \frac{{|∆F|}^{2}}{\langle {|∆F|}^{2}\rangle }\right)}^{-1}$$

for structure factor amplitude differences Δ*F* (short for Δ*F*_hkl_) and their errors σ_Δ*F*_, with angled brackets indicating averages over Miller indices. Using the same refinement procedure, dark and 2-ms models were refined for Fig. 2. Local scaling was performed using a custom script available in the Zenodo deposition and SCALEIT (CCP4 software suite version 7.1) was run from rs-booster version 0.1.2 (https://github.com/rs-station/rs-booster) as rs.scaleit (see Supplementary text S7).

Difference maps were visualized in PyMOL version 2.5.2 (*91*). Data processing scripts can be found in the Zenodo deposition.

### Lysozyme data collection

NaI-soaked lysozyme crystals were prepared as described previously (*92*). Monochromatic data were collected on the Northeastern Collaborative Access Team (NE-CAT) beamline 24-ID-C (Advanced Photon Source, Argonne National Laboratory) on 12 August 2020. Diffraction data were collected at ambient temperature (about 295 K). Three 1440-image passes (720°) were collected from a single crystal of lysozyme with an exposure time of 0.1 s and an oscillation angle of 0.5°. The incident x-ray intensity, at an energy of 11.95 keV, was attenuated to 0.5% transmission, which corresponds to an estimated flux of 2.4 × 10^10^ photons s^−1^. Data were collected using helical acquisition, so that dose was evenly distributed along the crystal. The PILATUS 6M-F detector (Dectris) was positioned at the minimal distance of 150 mm.

Laue data were collected on the same day at BioCARS (Advanced Photon Source, Argonne National Laboratory), similar to data collection described in previous work (*29*), except that Laue stills were collected in 1° steps. Data were collected at ambient temperature (about 295 K).

### Thermolysin and lysozyme

Careless 0.4.1 was run with a bivariate prior, sweeping over 15 double-Wilson *r* values on two datasets, one of thermolysin containing 3160 images collected from a serial XFEL experiment, CXIDB 81 (*68*) and one of lysozyme containing the first 720 images from the above data collection. Lysozyme data were indexed and integrated using Precognition (Renz Research Inc.) and Laue-DIALS version 0.4 (*66*) (https://github.com/rs-station/laue-dials). Thermolysin unmerged intensities (in DIALS .pickle format) were obtained from CXIDB 81 (www.cxidb.org/id-81.html). Both datasets were split according to Friedel symmetry and scaled and merged in Careless, applying the bivariate prior to relate anomalous half datasets. In the case of thermolysin, direct optimization of *r* was done in Careless 0.5.3. Next, the anomalous half datasets were combined into single datasets of merged structure factor amplitudes. Correlation coefficients on these datasets were computed using careless.cchalf, careless.ccpred, and careless.ccanom (*63*). For lysozyme, we used Epinorm (Renz Research Inc.) as a benchmark for scaling and merging. The scaling benchmark for thermolysin (using cctbx.xfel version 2021.11.dev3 + 4.g05389c3054) was described previously (*54*). Phenix version 1.20.1 (*89*) was used to phase the resultant structure factor amplitudes by isomorphous replacement with PDB ID 2TLI in the case of thermolysin and with a high-resolution monochromatic model in the case of lysozyme (table S1). In addition, an anomalous omit map was phased. The phases and omit map were then used for visualization of anomalous omit peaks with PyMOL and measurement of peak heights with reciprocalspaceship. Data processing scripts can be found in the Zenodo deposition.

### Fragment screening of Mac1

Data collection and analysis of a crystallographic drug fragment screen of SARS-CoV-2 Nsp3 Mac1 have been previously described (*14*). For the work described here, 1 unmerged apo. dataset and 16 unmerged holo HKL files (previously processed using XDS (X-ray Detector Software) version 31 January 2020) were converted to mtz files and then processed with Careless version 0.4.1, with use of a univariate or multivariate prior to relate the apo. and 16 holo datasets. The Bayesian network was trained following fig. S14, with dependencies drawn between the apo. dataset and each of the 16 holo datasets (see Supplementary Text). In addition, we introduced a control apo. dataset, a duplicate of the reference that also depended on the reference apo. dataset, and difference maps were computed by subtracting each holo dataset from the control apo. dataset. To compensate for the duplication of the reference apo. dataset, we multiply the errors of the intensities by $\sqrt{2}$. The double-Wilson *r* was set to a uniform value for all 16 apo-holo dependencies and was varied between Careless runs as 1 to 0.5^run number^. Metadata keys included observed Miller indices, detector position and positional encoding, image number, the azimuthal angle PSI (*93*), diffracted beam direction parameters ALF1 and BET1 (*94*), and dataset one-hot encoding. The PSI angle, ALF1, and BET1 metadata keys are from integration in XDS. To perform one-hot encoding of dataset IDs, one metadata key is created for every dataset. Each reflection receives the value 1 in the corresponding dataset column and elsewhere 0. One-hot encoding was implemented using a reciprocalspaceship script. Correlation coefficients were computed using careless.ccpred. Apo. phases were obtained by refinement of a model against the apo. amplitudes scaled using a univariate prior. Refinement was performed using PHENIX version 1.20.1. The apo. phases were then used for creating isomorphous difference maps of each holo minus apo. dataset. We created difference maps and calculated peak heights and real-space correlation coefficients using rs-booster version 0.1.1 (https://github.com/rs-station/rs-booster). To compute an *F*_c_ − *F*_c_ map, we used phenix.fmodel to compute *F*_c_ values from PDB ID 7KQO as the apo. model and PanDDA models as the holo models. We then scaled the *F*_c,holo_ to the *F*_c,apo_. PanDDA models, 1-BDC values, and *Z* maps were taken from an analysis that included all previously reported datasets (*14*, *72*). Data processing scripts can be found in the Zenodo deposition.

## Supplementary Materials

### This PDF file includes:

Supplementary text Figs. S1 to S19 Tables S1 and S2 References
