# Supplementary material for: Sensitive detection of structural dynamics using a statistical framework for comparative crystallography
Source: Sci Adv. 2025 Dec 3;11(49):eadj2921. doi: 10.1126/sciadv.adj2921 (PMC12674120; doi:10.1126/sciadv.adj2921)
Supplement: Supplementary file 1 — Supplementary text Figs. S1 to S19 Tables S1 and S2 References [file sciadv.adj2921_sm.pdf]

Science Advances
AAAS

Supplementary Materials for

Sensitive detection of structural dynamics using a statistical framework for comparative crystallography

Doeke R. Hekstra *et al.*

Corresponding author: Doeke R. Hekstra, doeke_hekstra@harvard.edu;
Kevin M. Dalton, kmdalton@slac.stanford.edu

Sci. Adv. **11**, eadj2921 (2025)
DOI: 10.1126/sciadv.adj2921

This PDF file includes:

Supplementary text
Tables S1 and S2
Figs. S1 to S19
References

Supplementary Text

S1. Scaling

X-ray diffraction images obtained from macromolecular crystals consist of patterns of many small spots, or reflections. The true intensities of these reflections are proportional to the square amplitudes of the "structure factors"—the Fourier components of the electron density in the crystal. The observed intensities, however, also depend on a range of multiplicative factors ("scales") due to the intensity and polarization of the incident beam, the volume of crystal exposed to the beam, intrinsic and beam-induced crystal defects, and absorption of the beam by the surrounding material, air, and the detector itself. Neither the true reflection intensities nor these scales are directly observed, yet it is critical that before inferring changes in structure from changes in observed intensities, these intensities must be corrected. The correction procedure is known as scaling. A comparison with the election forecasts is instructive: raw counts from opinion polls need to be corrected for differences in the rates at which different groups of voters (photons) respond to surveys (are detected). Such corrections (scales) are typically estimated from the characteristics of those who respond (metadata), while errors in these corrections contribute to overall non-sampling error (*105*). Inaccurate corrections for non-response bias have contributed to prominent forecasting errors in US presidential elections (*106, 107*).

Approaches to scaling of crystallographic data primarily rely on comparison of redundant observation of equivalent reflections which should yield the same structure factor amplitude up to variation in the scale factor. Sample and instrument time are typically highly limited. As a result, the need for scaling to compare different conditions generates a tradeoff: one can either accurately estimate scales by maximizing redundancy for a few conditions or observe many conditions but be left with residual systematic differences in the scales of each dataset. These remaining scaling inaccuracies are often addressed after the fact, for example in SOLVE (*108*), SCALEIT (*104*), or using the "Isomorphous Difference Map" utility of PHENIX, e.g., in (*44, 109*).

S2. A statistical model for correlations of related structure factors

S2.1 The Wilson distribution

The Wilson distribution provides a simple, long-standing model for the statistical distribution of the structure factors of macromolecular crystals (*57, 110*). Briefly, structure factors can be calculated as a sum over contributing atoms, j, in the complex plane, that is,

$$\boldsymbol{F}_h = \sum_j f^B_{\boldsymbol{h}j} e^{2\pi i(\boldsymbol{h}^T \boldsymbol{x}_j)} \quad [1]$$

where $f^B_{\boldsymbol{h}j}$ captures the finite size of atoms and their thermal disorder, and $\boldsymbol{h}^T\boldsymbol{x}_j$ is the dot product of the atomic fractional coordinate vector $\boldsymbol{x}_j$ and Miller indices $\boldsymbol{h}$. (We will use boldface notation for vectors and the complex structure factors $\boldsymbol{F}_h$, while reserving italics for structure factor amplitudes $F_h \equiv |\boldsymbol{F}_h|$ and other scalar quantities.) For most reflections, as each subsequent atom is added to the calculation, the growing structure factor performs a nearly random walk in the 2D complex plane, and the central limit theorem can be applied(*57*). The rest of the reflections are centric and thus constrained to lie on a line through the origin due to crystallographic symmetry restrictions (*57*). Thus, the acentric structure factors approximately follow a bivariate normal,

while centrics follow a univariate normal distribution, both with zero mean. To illustrate, for the real and imaginary components of an acentric structure factor $Re(\boldsymbol{F_h})$ and $Im(\boldsymbol{F_h})$ respectively,

$$P(Re(\boldsymbol{F_h}), Im(\boldsymbol{F_h})) = \mathcal{N}\left(0, \frac{1}{2}\epsilon_{\boldsymbol{h}}\Sigma_{\boldsymbol{h}} I\right) \quad [2]$$

where the multiplicity ϵ_h accounts for crystallographic symmetry(*57*), I is a 2x2 identity matrix, and $\Sigma_{\boldsymbol{h}}$ is an overall scale. In the Wilson model, $\Sigma_{\boldsymbol{h}}$ represents the total scattering power of atoms in the unit cell as a function of resolution (that is: $\mathbb{E}(|\boldsymbol{F_h}|^2) = \epsilon_{\boldsymbol{h}}\Sigma_{\boldsymbol{h}} = Re(\boldsymbol{F_h})^2 + Im(\boldsymbol{F}_h)^2$. It will be convenient to normalize structure factors $\boldsymbol{E_h} = \frac{\boldsymbol{F_h}}{\sqrt{\epsilon_{\boldsymbol{h}}\Sigma_{\boldsymbol{h}}}}$, such that

$$P(Re(\boldsymbol{E_h}), Im(\boldsymbol{E_h})) \sim \mathcal{N}\left(0, \frac{1}{2}I\right) \quad \text{for acentric structure factors and} \quad [3a]$$

$$\boldsymbol{E_h} \sim \mathcal{N}(0,1) \quad \text{for centric structure factors.} \quad [3b]$$

For initial inspection of already-merged data, we will treat Σ as an empirical normalization constant (**Supplementary Notebooks 1-3,6**). When scaling and merging diffraction data, we note that *Careless* treats Σ as the scale function that needs to be inferred.
In X-ray crystallography the phase of the structure factor is not observed (*111*), so the distribution of the amplitudes of structure factors (here denoted $E_{\boldsymbol{h}}$) is of great interest. Following Wilson (*57*), this distribution can be obtained by first converting from Cartesian to polar coordinates and then integrating over the unknown phases to yield the standard Wilson distribution,

$$P(E_{\boldsymbol{h}}) = \begin{cases} \sqrt{\frac{2}{\pi}}\exp\left(-\frac{1}{2}E_{\boldsymbol{h}}^2\right) & \text{for } \boldsymbol{h} \text{ centric} \\ 2E_{\boldsymbol{h}}\exp(-E_{\boldsymbol{h}}^2) & \text{for } \boldsymbol{h} \text{ acentric} \end{cases} \quad [4]$$

This distribution is the basis for many models of structure factor amplitudes(*110*).

S2.2 The bivariate Wilson distribution

When considering a pair of data sets collected under similar conditions, we treat the sum in eq. [1] for two data sets as correlated random walks (*58*) with correlation coefficients, r, between the corresponding real components, and likewise between imaginary components (**Figure 1a**). In this approach, the joint probability distribution of two acentric structure factors $\boldsymbol{E}_{\boldsymbol{h}}^A$ and $\boldsymbol{E}_{\boldsymbol{h}}^B$ is (*58*):

$$P\left(Re(\boldsymbol{E}_{\boldsymbol{h}}^A), Im(\boldsymbol{E}_{\boldsymbol{h}}^A), Re(\boldsymbol{E}_{\boldsymbol{h}}^B), Im(\boldsymbol{E}_{\boldsymbol{h}}^B)\right) = \mathcal{N}\left(0, \frac{1}{2}\begin{bmatrix} 1 & 0 & r & 0 \\ 0 & 1 & 0 & r \\ r & 0 & 1 & 0 \\ 0 & r & 0 & 1 \end{bmatrix}\right) \quad [5]$$

with the parameter r henceforth called the "double-Wilson r". The conditional probability distribution of $\boldsymbol{E}_{\boldsymbol{h}}^B$ given $\boldsymbol{E}_{\boldsymbol{h}}^A$ can now be calculated as $P\left(Re(\boldsymbol{E}_{\boldsymbol{h}}^B), Im(\boldsymbol{E}_{\boldsymbol{h}}^B)|\boldsymbol{E}_{\boldsymbol{h}}^A\right) = \mathcal{N}\left(r\boldsymbol{E}_{\boldsymbol{h}}^A, \frac{1}{2}(1-r^2)I\right)$, and the conditional probability distribution of their amplitudes (i.e. $E_{\boldsymbol{h}}^B$ conditional on $E_{\boldsymbol{h}}^A$) can be calculated by marginalizing over both the unknown phase of $\boldsymbol{E}_{\boldsymbol{h}}^A$ and the unknown phase difference between $\boldsymbol{E}_{\boldsymbol{h}}^A$ and $\boldsymbol{E}_{\boldsymbol{h}}^B$(*59*), yielding the Rice distribution,

$$P(E_h^B) \sim Rice(\nu, \sigma^2) = \frac{x}{\sigma^2} \exp\left(\frac{-(x^2+\nu^2)}{2\sigma^2}\right) I_0\left(\frac{x\nu}{\sigma^2}\right)$$

with $x = E_h^B$ and parameters $\nu = rE_{\boldsymbol{h}}^A$ and $\sigma^2 = \frac{1}{2}(1-r^2)$ and I_0 the modified Bessel function of the first kind with order zero. This derivation is elaborated upon in **Supplementary Notebook 5**.

Now for centric structure factors $\boldsymbol{E}_{\boldsymbol{h}}^A$ and $\boldsymbol{E}_{\boldsymbol{h}}^B$ we have:

$$P(\boldsymbol{E}_h^A, \boldsymbol{E}_h^B) = \mathcal{N}\left(0, \frac{1}{2}\begin{bmatrix} 1 & r \\ r & 1 \end{bmatrix}\right)$$

so that the probability distribution of their amplitudes $E_{\boldsymbol{h}}^B$ conditional on $E_{\boldsymbol{h}}^A$ is

$$P\left(E_{\boldsymbol{h}}^B | E_{\boldsymbol{h}}^A\right) = \text{FoldedNormal}(\mu, \sigma^2) = \frac{1}{\sqrt{2\pi\sigma^2}}\left(\exp\left(-\frac{(x-\mu)^2}{2\sigma^2}\right) + \exp\left(-\frac{(x+\mu)^2}{2\sigma^2}\right)\right)$$

for $x = E_h^B$, the magnitude of $\boldsymbol{E}_{\boldsymbol{h}}^{\boldsymbol{B}}$, and parameters $\mu = rE_{\boldsymbol{h}}^A$ and $\sigma^2 = (1-r^2)$.

Following ref. (*58*), we note that r often depends on resolution, consistent with a model proposed by Luzzati, such that $r \approx ae^{-bs^2}$, where s is 1/resolution. We also note that r plays a role analogous to σ_A in crystallographic refinement (*58, 112*) and can be estimated similarly. To illustrate this, we fit related pairs of structure factor amplitudes to this model, obtaining estimates of a and b, in "**Estimation of r from merged structure factor amplitudes"** and in **Supplementary Notebook 3**. We observe that a pair of synthetic datasets obtained by resampling of example observed data (while imposing $r = ae^{-bs^2}$) yields a resolution-dependent correlation (blue line in **Figure S18**) similar to the observed correlation (green line in **Figure S18**). The inferred true correlations between structure factor amplitudes (dashed magenta line in **Figure S18**) is of similar magnitude, indicating most of the decay in correlation with resolution is real rather than due to measurement errors (see also **Supplementary Notebook 2b**).

S2.3 The multivariate Wilson distribution

The conditional independence graphs in **Figures 7a** and **S13** all take the form of a tree (a connected, acyclic graph). Given this, it is straightforward to calculate the full covariance matrix for the joint distribution of complex structure factors from the diagram (*62*) (**Supplementary Notebook 7**). More importantly, we can factorize the joint probability of structure factors as

$$P\left(\boldsymbol{E}_{\boldsymbol{h}}^{(1)}, \boldsymbol{E}_{\boldsymbol{h}}^{(2)}, \boldsymbol{E}_{\boldsymbol{h}}^{(3)}, \ldots\right) = P\left(\boldsymbol{E}_{\boldsymbol{h}}^{(0)}\right) \cdot \prod_{j>0} P\left(\boldsymbol{E}_{\boldsymbol{h}}^{(j)} \middle| \boldsymbol{E}_{\boldsymbol{h}}^{\mathrm{Pa}(j)}\right) \qquad [6a]$$

where we set the index of the root of the tree to 0, and $\mathrm{Pa}(j)$ is the "parent node" of node j. Whenever this is possible, we can integrate over phase differences factor by factor, and obtain an analogous expression for structure factor amplitudes,

$$P\left(E_{\boldsymbol{h}}^{(1)}, E_{\boldsymbol{h}}^{(2)}, E_{\boldsymbol{h}}^{(3)}, \ldots\right) = P\left(E_{\boldsymbol{h}}^{(0)}\right) \cdot \prod_{j>0} P\left(E_{\boldsymbol{h}}^{(j)} \middle| E_{\boldsymbol{h}}^{Pa(j)}\right). \qquad [6b]$$

For acentric reflections, $P\left(E_{\boldsymbol{h}}^{(j)} \middle| E_{\boldsymbol{h}}^{Pa(j)}\right)$ again follows a Rice distribution, now with $\nu = r_j E_{\boldsymbol{h}}^{Pa(j)}$ and $\sigma^2 = \frac{1}{2}\left(1 - r_j^2\right)$, with r_j as the double-Wilson r for the correlation between node j and its parent. For centric reflections, $P\left(E_{\boldsymbol{h}}^{(j)} \middle| E_{\boldsymbol{h}}^{Pa(j)}\right)$ again follows a folded normal distribution with $\mu = r_j E_{\boldsymbol{h}}^{Pa(j)}$ and $\sigma^2 = \left(1 - r_j^2\right)$. We provide a numerical demonstration of equation [6b] in **Supplementary Notebook 7**. We will refer to the distribution in eq. [6b] as the *multivariate Wilson distribution*.

S3 Using Careless

The Careless software package can be installed following the instructions on this GitHub page: https://github.com/rs-station/careless. Careless comes with various examples at https://github.com/rs-station/careless-examples for use of univariate priors. The options, flags, and hyperparameter settings implemented in Careless, as well as broad recommendations for use of Careless, are explained in ref. (*63*). We additionally discuss use of Careless in the below text.

Use of Careless implicitly involves optimizing a model, which we now discuss briefly. Careless estimates (1) the parameters of a multilayer perceptron (neural network) that itself estimates scales from the metadata provided by the user, and (2) location and scale parameters for the posterior distribution of structure factor amplitudes. To do so, Careless minimizes an objective function subject to a set of hyperparameters. The double-Wilson *r* is such a hyperparameter, as it controls the properties of the prior distribution of structure factor amplitudes. Other hyperparameters specify the use of positional encoding (providing transformations of the provided metadata to make machine learning more efficient) and the number of degrees of freedom of the error model when using a Student *t* distribution (for data with many outlier observations, values in the range from 1-32 are good; otherwise we recommend values of 64 or greater). In accompanying work (*56*), we systematically evaluate the effect on difference density and validation statistics from several "sweeps" over Careless hyperparameters. We additionally point the user to the `dw-examples` directory at https://github.com/Hekstra-Lab/dw-examples for annotated examples of use of a multivariate prior with Careless. For each example, in the `careless_runs` subfolder, we provide hyperparameter sweep scripts implemented for the job manager Slurm (*113*).

Aside from the recommended hyperparameter sweeps in (*56*), Careless also provides two hyperparameters related to the neural network architecture, `--mlp-width` and `--mlp-layers`, as well as one relating to training, `--mc-samples`. These are described in the Careless `-h` documentation and we now provide several recommendations for their settings.

1. We do not recommend changing `--mlp-layers`, the number of dense layers of the multilayer perceptron used for estimating the scale function.

2. We recommend the largest `--mlp-width` possible that does not exceed the number of metadata keys plus 4 times the number of positional encoding keys. The default setting of `--mlp-width` is 10, but we have found in our experience that this produces worse results than a manually-set `--mlp-width` according to the above guidelines.

3. If the objective function trace looks choppy in `careless.loss_history` (there are tall peaks as the optimization proceeds), we recommend using more `--mc-samples` than the default 1. `--mc-samples` is the number of Monte Carlo samples to take per estimate of the ELBO objective function.

S4 Using the multivariate Wilson model

In Careless 0.4.1, the multivariate Wilson model is defined with two flags, `--double-wilson-parents` and `--double-wilson-r`, whose syntax is explained in the dw-examples GitHub repository (*74*) and in (*63*). As of Careless version 0.5.3, Careless also supports direct, internal optimization of the double-Wilson r parameter.

Guidelines for how to structure the dependencies between datasets are presented in **Figure S14** and presented here for the fragment screening dataset: first, we considered how to draw the dependencies between nodes in a Bayesian network. We reasoned that the *apo* dataset represents an unperturbed state and that the candidate *holo* datasets represent a distinct perturbation of the *apo* dataset, which means that we can draw dependencies from the *apo* dataset to each *holo* dataset. To control for any spurious effects of dependencies, we recommend adding a control *apo* dataset that depends on the parent *apo* dataset. We then generated *holo* minus *apo* difference maps using this control dataset.

The other cases presented in the text are all simpler: when two conditions (or, for anomalous signal, sets of Miller indices) are compared, the choice of parent node and child node is arbitrary because the implied correlations are the same regardless of this choice. We note that cross-validation statistics sometimes exhibit artificially high values for r above 0.999 and real-space or structure refinement metrics should serve as the final guide on selection of r.

We generally anticipate the user has an unperturbed, control sample that can serve as the reference. But even without such a dataset, use of a multivariate prior is likely useful. One could either compare a few choices for the root of the dependency graph to check for robustness of the results, or add a "dummy" root node with high variance, so as to not influence the scale and amplitude estimation, but to retain the correlation between the datasets (see ref. (*114*) for an analogous approach to multiple isomorphous replacement). In the drug fragment screening example, use of a dummy node results in little to no loss of performance (**Figure S16e**).

To help users discern whether a multivariate prior is appropriate for their data, we direct the user to the examples in **Figures 2** and **S3-S5**: datasets need not be acquired from protein of the same batch, nor does the data collection hardware or data processing software (up until scaling and merging) need to be the same. Nevertheless, the quality of comparative crystallography experiments is often determined by the similarity of the systematic errors in constituent datasets.

Consequently, the benefits of joint scaling will likely improve if datasets are more closely related. Correction for systematic errors will be most efficient if these errors are correlated between the datasets, e.g. the same beam and detector are used. There are two hard constraints. First, datasets should be isomorphous or near-isomorphous, such that structure factors will be correlated (12). Second, related datasets must be formatted with consistent metadata.

S5 Estimation of *r* from merged structure factor amplitudes

The double-Wilson parameter *r* is closely related to the correlation between two datasets. In **Supplementary Notebook 2a**, we find numerically that the Pearson correlation between two synthetic datasets of normalized structure factor amplitudes, E_1 and E_2, is approximately r^2. Indeed, within the multivariate Wilson model, the *squares* of the structure factor amplitudes are correlated exactly as r^2 (*61*). Using the former relation, we may obtain a resolution-independent value for the double-Wilson r, but we may also fit the double-Wilson *r* as a function of resolution. To do so, we model variation in the amplitudes as follows. We model $E_{1,obs} = x + \eta_1$, where x is the true value of E_1 and η_1 is measurement error. We model the second dataset as $E_{2,obs} = x + \epsilon + \eta_2$, where ϵ is the true difference between E_2 and E_1. We assume that x, ϵ, η_1 and η_2 are uncorrelated with variances σ_x^2, σ_ϵ^2, σ_1^2 and σ_2^2. Then, we have

$$\rho_{obs} = \rho(x + \eta_1, x + \epsilon + \eta_2) = \frac{\sigma_x^2}{\sqrt{(\sigma_x^2 + \sigma_1^2)(\sigma_x^2 + \sigma_\epsilon^2 + \sigma_2^2)}}$$

and

$$\rho_{true} = \rho(x, x + \epsilon) = \frac{\sigma_x^2}{\sqrt{(\sigma_x^2)(\sigma_x^2 + \sigma_\epsilon^2)}}$$

so that

$$\rho_{obs}^{-2} = \rho_{true}^{-2} + \frac{\sigma_1^2 + \sigma_2^2}{\sigma_x^2} + \frac{\sigma_1^2\sigma_2^2}{\sigma_x^4} + \frac{\sigma_1\sigma_2}{\sigma_x^2}(\rho_{true}^{-2} - 1)$$

σ_x^2 is a known property of the Wilson distribution, allowing us to relate the true correlation between E_1 and E_2 to the observed correlation in the presence of measurement errors. With this relationship in hand, we can then find the value of r by approximating $\rho_{true} \approx r^2$ and, if modeling resolution-dependence, of a, b such that $r = a \cdot e^{-bs^2}$.

In practice, it is difficult to estimate r to the precision needed, but we can get close. Correlation coefficients from merged MTZ files result in *r*=0.993, 0.996, and 0.912 in the lysozyme, PYP, and thermolysin examples, respectively, while the optimal *r* is 0.999, 0.9995, and 0.996.

S6 Normalizing structure factors

The structure factor amplitudes used for the examples in **Figures 3** and **S2-5** were obtained from datasets that were processed using DENZO/SCALEPACK (*115*), XSCALE (*94*), or SCALA

(*65*). The structure factor amplitudes F_h produced by these software packages are not normalized. To compare with our statistical model, and remove residual systematic errors in scaling, we normalized the $F_{\boldsymbol{h}}$ as described in **Supplementary Notebook 1** and **1a**. To do so, we introduced an ad hoc normalization procedure (which is not relevant to the results in **Figures 4-7**, which were obtained by scaling and merging using Careless). To this end, we first observe that the Wilson model can be formulated more generally as

$$P(F_{\boldsymbol{h}}) = \begin{cases} \sqrt{\dfrac{2}{\pi \epsilon_{\boldsymbol{h}} \Sigma_{\boldsymbol{h}}}} \exp\left(-\dfrac{F_{\boldsymbol{h}}^2}{2\epsilon_{\boldsymbol{h}}\Sigma_{\boldsymbol{h}}}\right) & \text{for } \boldsymbol{h} \text{ centric} \\ 2\dfrac{F_{\boldsymbol{h}}}{\epsilon_{\boldsymbol{h}}\Sigma_{\boldsymbol{h}}} \exp\left(-\dfrac{F_{\boldsymbol{h}}^2}{\epsilon_{\boldsymbol{h}}\Sigma_{\boldsymbol{h}}}\right) & \text{for } \boldsymbol{h} \text{ acentric} \end{cases}$$

where $\epsilon_{\boldsymbol{h}}$ is the multiplicity of reflections, and $\Sigma_{\boldsymbol{h}}$ dictates the mean square intensity of reflections at (or nearby) Miller index $\boldsymbol{h}$ (short for (h, k, l)). To use Wilson statistics, we must learn how Σ varies across reciprocal space—or, equivalently, infer normalized structure factor amplitudes $E_{\boldsymbol{h}} = \frac{F_{\boldsymbol{h}}}{\sqrt{\epsilon_{\boldsymbol{h}}\Sigma_{\boldsymbol{h}}}}$, which obey $\langle E^2 \rangle = 1$ and follow the Wilson distribution for normalized structure factors.

In **Supplementary Notebook 1**, we normalize structure factors in three steps. First, we find an optimal anisotropic *B* matrix that best approximates a standard Wilson distribution. That is, we model $\Sigma_{\boldsymbol{h}}$ as $\Sigma'_{\boldsymbol{h}} = a \cdot \exp\left(-\frac{1}{2}Bs_{\boldsymbol{h}}^2\right)$, with $s_{\boldsymbol{h}} = \frac{1}{d_{hkl}}$, and find the maximum of the likelihood function $l(a, B) = P(\{F_{\boldsymbol{h}}\} \mid a, B) = \prod_{\boldsymbol{h}} P(F_{\boldsymbol{h}} \mid a, B)$. Such likelihood maximization under the Wilson distribution has been previously described (*116, 117*).
At this point, the mean squared intensity is often still not uniform across reciprocal space. This is for several reasons, such as incomplete correction for absorption artifacts during scaling, as well as the occurrence of regularities in protein structure (e.g. secondary structure). A heuristic to correct for this is to propose that $\Sigma_{\boldsymbol{h}}$ be modulated by position in reciprocal space as:

$$\Sigma''_{\boldsymbol{h}} = \left(\sum_{\boldsymbol{n}} A_{\boldsymbol{n}} \cos\left(\frac{2\pi \boldsymbol{h}^T \boldsymbol{n}}{L}\right) + B_{\boldsymbol{n}} \sin\left(\frac{2\pi \boldsymbol{h}^T \boldsymbol{n}}{L}\right)\right) \Sigma'_{\boldsymbol{h}}$$

with the anisotropically determined value $\Sigma'_{\boldsymbol{h}}$, the Fourier component $\boldsymbol{n} \in [0, 1, 2, 3, 4]^{\boldsymbol{3}}$, and the entire sum computed in a box with side lengths L that bounds the observed reciprocal lattice points. We then find the optimal $A_{\boldsymbol{n}}$ and $B_{\boldsymbol{n}}$ that minimize the loss between the resultant $F'_{\boldsymbol{h}} = \frac{F_{\boldsymbol{h}}}{\sqrt{\epsilon_{\boldsymbol{h}}\Sigma''_{\boldsymbol{h}}}}$ and the standard Wilson distribution, leaving out 15% of the reflections for cross-validation to determine the optimal maximal value of components in $\boldsymbol{n}$.

Finally, we locally estimate $\Sigma'''_{\boldsymbol{h}} = \frac{\mathbb{E}(|\boldsymbol{F_h}|^2)}{\epsilon_{\boldsymbol{h}}}$ using *k*-nearest neighbor (KNN) estimation of $\mathbb{E}(|\boldsymbol{F_h}|^2)$ in reciprocal space, where $\epsilon_{\boldsymbol{h}}$ denotes the multiplicity as before. Starting from the structure factor amplitudes $F'_{\boldsymbol{h}}$ in the previous step, we compute $\Sigma'''_{\boldsymbol{h}}$ across 50-1,600 neighbors.

We choose the optimal kernel size by cross-validation. We do a final scaling $E_{\boldsymbol{h}} = \frac{F'_{\boldsymbol{h}}}{\sqrt{\epsilon_{\boldsymbol{h}} \Sigma'''_{\boldsymbol{h}}}}$ to arrive at approximately normalized structure factors distributed according to the standard Wilson distribution (**Figure S19**).

S7 Partial excitation & scale adjustment after merging

Perturbation-induced ON-OFF difference maps often resemble a negative image of the OFF electron density. Indeed, the univariate PYP difference map is dominated by the negative OFF difference density (**Figures 5c** and **S10b, left**). The effects of perturbations, whether due to laser excitation (*7*), temperature (*28, 118*), electric field (*29*), or small-molecule binding (*14*), often only affect a fraction of the molecules in the crystal. As a consequence, the resulting mixed structure factor amplitudes tend to be smaller than the ground-state (OFF) amplitudes (*119*), and the OFF electron density is more prominent in the resulting ON-OFF difference maps. It is common practice to rescale merged data to ensure similar average magnitude of OFF and ON structure factor amplitudes. In the developed statistical framework, this approach can be examined analytically.

Typically only a portion p of the protein molecules in a crystal is driven from the ground state (GS) into the one or more excited states (ES). Here, we examine a model of such partial excitation introduced by Coppens et al. (*119*). In this 'random distribution' model, the (complex) ON structure factors $\boldsymbol{F}^{on}$, i.e., the structure factors after perturbation is applied, depend on the ground-state structure factors $\boldsymbol{F}^{off} = \boldsymbol{F}^{gs}$ as well as the excited-state structure factors $\boldsymbol{F}^{es}$ so that

$$\boldsymbol{F}^{on} = p\boldsymbol{F}^{es} + (1-p)\boldsymbol{F}^{gs}$$

Under the assumptions described above (see **The bivariate Wilson distribution**), we observe that

$$\mathbb{E}(\boldsymbol{F}^{on}) = (1 - p + p \cdot r)\boldsymbol{F}^{gs} < \boldsymbol{F}^{gs},$$

since $p \in [0,1)$ and $r < 1$, such that, on average, ON amplitudes are slightly smaller than OFF amplitudes. To sensitively measure the effect of a perturbation on a crystal, often a difference map is constructed by subtracting related structure factor amplitudes, here F^{on} and F^{off}, and combined with the OFF phases. As a result, naïve ON-OFF difference maps can be negatively correlated with the ground-state density. It is common to scale the related datasets to each other, after merging each separately, using tools like SCALEIT (*104*) or local scaling (*103*). With the multivariate Wilson model, we can analyze the effects of this choice on the difference map bias and ground-state correlation. In **Supplementary Notebook 6**, we compute two statistics to assess the quality of the scaled difference map $\boldsymbol{\Delta F}_{obs} = (kF^{on} - k'F^{off})\exp(i\varphi^{off})$, where φ^{off} is the phase of the ground state: (1) the mean squared error between the scaled difference map and the true difference map $\boldsymbol{\Delta F}_{\boldsymbol{true}} = \boldsymbol{F}^{es} - \boldsymbol{F}^{gs}$ averaged over the unit cell, and (2) the covariance between the scaled difference map and the ground-state electron density.

We find that placing the ON and OFF structure factors on the same scale (e.g., by choosing $\frac{k}{k'}$ as the median ratio of F^{off}/F^{on} or $\sum_h F^{off} / \sum_h F^{on}$) effectively minimizes the covariance with the OFF density while keeping mean squared map error low and correlation with the true difference map near its maximum (**Figure S11**), justifying the practice of scaling related structure factor amplitudes to each other after merging.

S8 Joint distributions of phases

Equation [5] also implies a statistical relationship between the phases of two data sets. Indeed, the difference of the calculated phases between refined *apo* and inhibitor-bound models follow the expected Von Mises distribution (*59, 120*)

$$P(\Delta\varphi|E_1, E_2) = \frac{1}{2\pi I_0(z)} \exp(z \cos(\Delta\varphi))$$

with

$$z = \frac{2E_1E_2}{(1-r^2)}$$

We illustrate a fit of this model to the obtained phase differences in **Figure S17**.

Supplementary Tables

	NaI-soaked lysozyme at 24-ID-C
**PDB ID**	9B7C
**number of passes**	3
**rotation (°)**	720
**Wavelength (Å)**	1.03752
**Resolution range (Å)**	56.15 - 1.101 (1.14 - 1.101)
**Space group**	P 43 21 2
**Unit cell**	79.41 Å, 79.41 Å, 37.84 Å, 90°, 90°, 90°
**Total reflections**	5,583,525 (44,497)
**Unique reflections**	85,431 (1,781)
**Multiplicity**	65.4 (13.1)
**Completeness (%)**	91.24 (36.20)
**Mean I/sigma(I)**	38.87 (1.44)
**Wilson B-factor (Å^2)**	14.17
**R_{merge}**	0.05847 (1.882)
**R_{meas}**	0.05887 (1.958)
**R_{pim}**	0.00674 (0.523)
**$CC_{1/2}$**	1.000 (0.480)
**CC***	1.000 (0.810)
**Mosaicity (°)**	0.064±0.001
**Figure of merit**	0.40
**Reflections used in refinement**	45,167 (1,762)
**Reflections used for R-free**	2,159 (81)
**R-work**	0.1153 (0.2215)
**R-free**	0.1267 (0.2231)
**CC_{work}**	0.973 (0.859)
**CC_{free}**	0.970 (0.889)
**CC_{anom}**	0.655 (0.070)
**Number of non-hydrogen atoms**	1,284
**Macromolecules**	1,176
**Ligands**	44
**Solvent**	86
**Protein residues**	129
**RMS(bonds) (Å)**	0.011
**RMS(angles) (°)**	1.05
**Ramachandran favored (%)**	99.21
**Ramachandran allowed (%)**	0.79
**Ramachandran outliers (%)**	0
**Rotamer outliers (%)**	0
**Clashscore**	2.1
**Average B-factor (Å^2)**	19.45
**macromolecules**	18.39
**ligands**	27.97
**solvent**	31.74

Table S1. Data collection, processing, and refinement statistics for the high-resolution monochromatic structure of NaI-soaked lysozyme. The refined model was used to phase the anomalous difference maps for **Figure 4**.

Dataset	PanDDA peak height (σ)	multivariate Wilson scaling peak height (σ)
**P0115**	35.29	37.41
**P0116**	8.41	8.72
**P0123**	14.45	7.67
**P0124**	20.72	16.64
**P0131**	9.61	16.06
**P0132**	19.73	15.92
**P0137**	19.02	16.65
**P0138**	28.94	24.72
**P0139**	13.79	14.24
**P0142**	6.96	6.32
**P0148**	11.18	13.16
**P0159**	37.16	38.41
**P0161**	41.49	45.87
**P0163**	19.21	14.48
**P0178**	26.14	48.31
**P0179**	20.88	21.74

Table S2. Comparison between PanDDA and multivariate Wilson analysis of the tallest ligand peak heights per dataset for the Mac1 fragment screening dataset.

Supplementary Figures

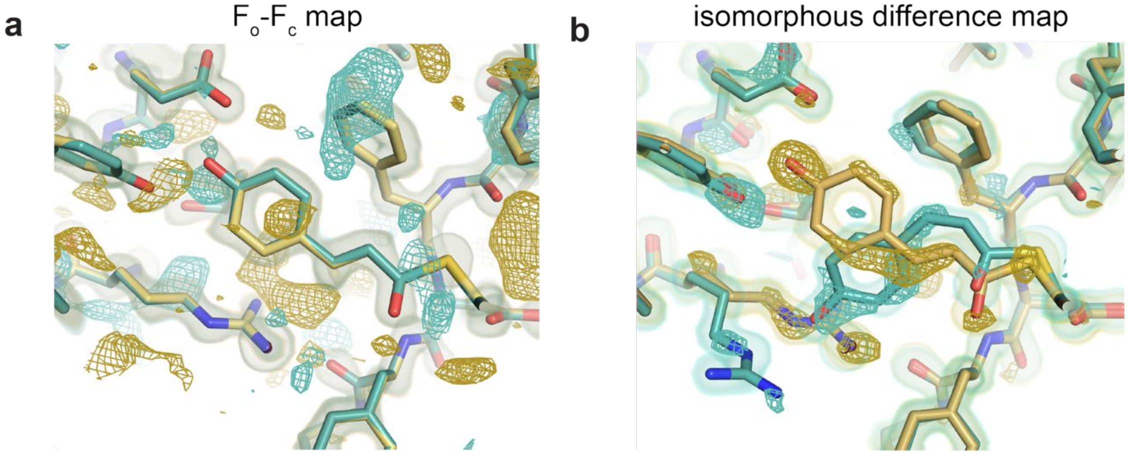

Figure S1. Low contour levels of F_o-F_c maps detect noise rather than conformational changes. a) Overlay of models and maps from **Figures 2c** and **2d**, along with the $F_o - F_c$ electron density of the 2ms map and model, a teal and yellow mesh contoured to +2σ and -2σ, respectively. There is little correspondence between the structural differences and the $F_o - F_c$ map. b) In contrast, the isomorphous 2ms−dark difference map (yellow and teal mesh, contoured to 3σ, unweighted, reproduced from **Figure 5c**), shows clear correspondence to the structural differences between 2ms and dark models (teal and yellow sticks, respectively). Also shown: calculated electron density at 2ms after excitation and in the dark state (teal and yellow surfaces, respectively; contoured to 1.5σ). Calculated structure factors from PDB entry 1TS0.

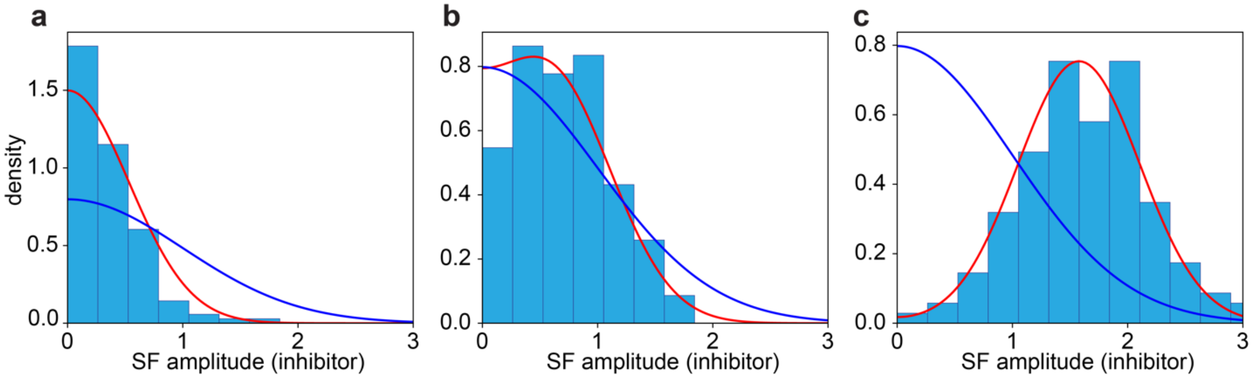

Figure S2. Conditional distributions describe centric structure factor amplitudes. Analogous to **Figure 3c-e**, histograms of *centric* structure factor amplitudes of PTP-1B in the presence of the TCS-401 inhibitor for reflections for which the structure factor amplitudes in the unliganded (apo) state fall within the **a)** 0-4th, **b)** 48-52th and **c)** 92-96th percentile. Red: folded normal distributions parametrized by the mean apo structure factor amplitude per bin, and $r_{DW} = 0.85$. Blue: centric Wilson distribution.

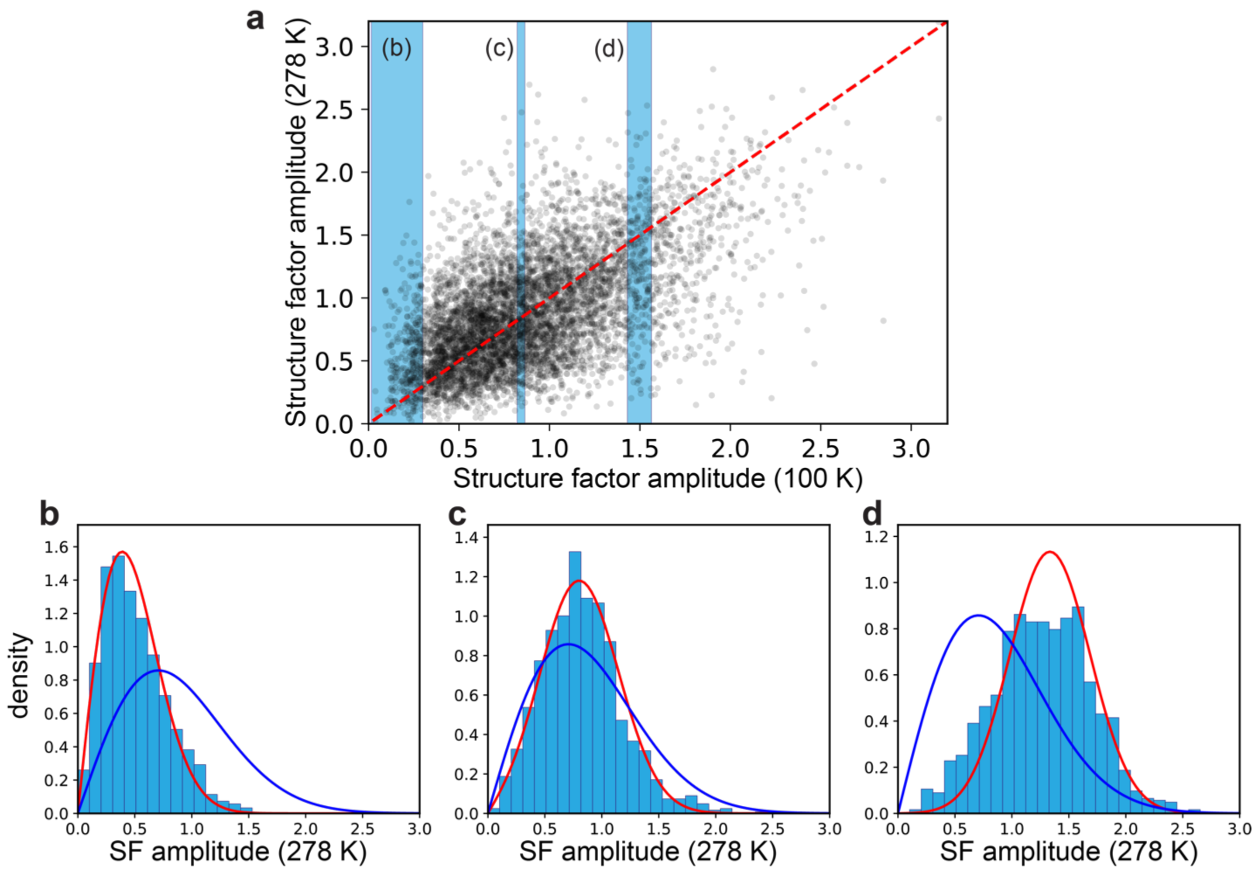

Figure S3. **The bivariate Wilson distribution for a pair of datasets across temperatures. a)** Scatter plot for a random subset of acentric structure factor amplitudes for two datasets of thaumatin, one measured at 100 K (PDB ID 5KVX) (*121*) and one measured at 278 K (PDB ID 5KW3) (*121*). Blue slices indicate data points for which histograms are shown in the next panels. **b-d)** Histograms for slices through shaded regions in panel **a** are better approximated by Rice distributions (red) parametrized by a double-Wilson r (here, r = 0.86) than by the Wilson distribution (blue).

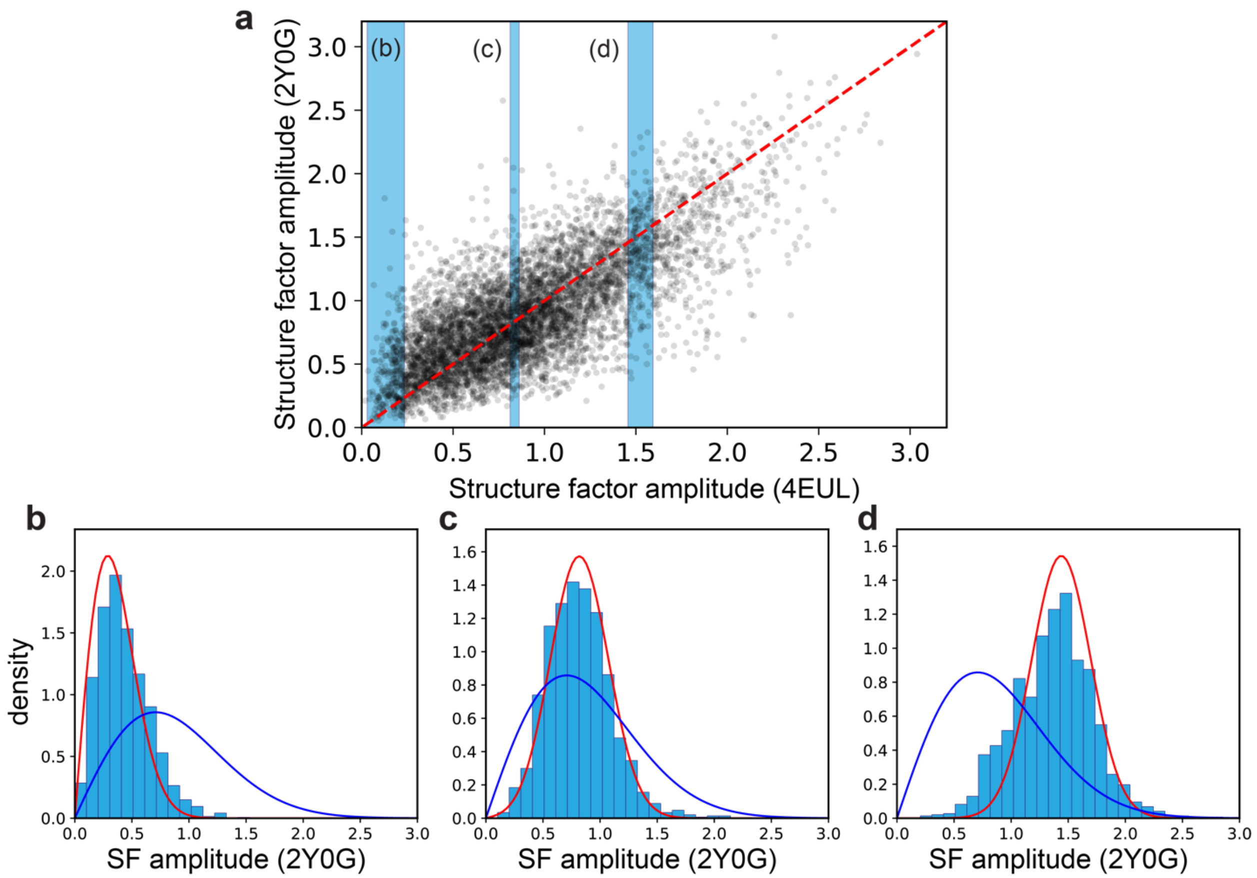

Figure S4. **The bivariate Wilson distribution for a pair of replicate datasets between labs. a)** Scatter plot for a random subset of acentric structure factor amplitudes for two datasets of eGFP, one reported in 2012 (PDB ID 4EUL) (*122*) and one reported in 2011 (PDB ID 2Y0G) (*123*). Blue slices indicate data points for which histograms are shown in the next panels. **b-d)** Histograms for slices through shaded regions in panel **a** are better approximated by Rice distributions (red) parametrized by a double-Wilson r (here, $r = 0.93$) than by the Wilson distribution (blue).

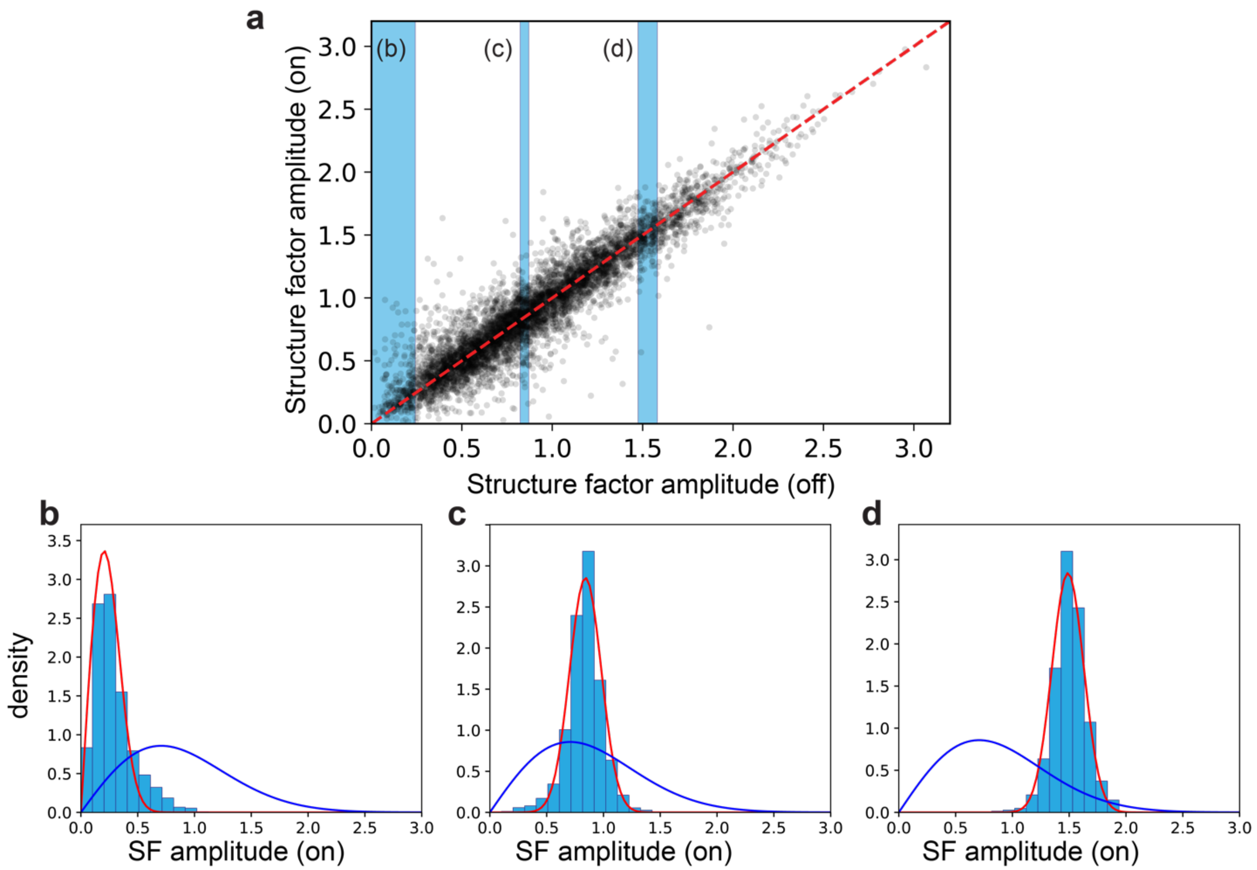

Figure S5. **The bivariate Wilson distribution for a pair of time-resolved datasets. a)** Scatter plot for a random subset of acentric structure factor amplitudes for two datasets of PYP, one measured without light illumination (PDB ID 1NWZ) (*124*) and one measured on a crystal cryotrapped after exposure to a 460 nm laser pulse (PDB ID 3PYP) (*125*). Blue slices indicate data points for which histograms are shown in the next panels. **b-d)** Histograms for slices through shaded regions in panel **a** are better approximated by Rice distributions (red) parametrized by a double-Wilson r (here, $r = 0.98$) than by the Wilson distribution (blue).

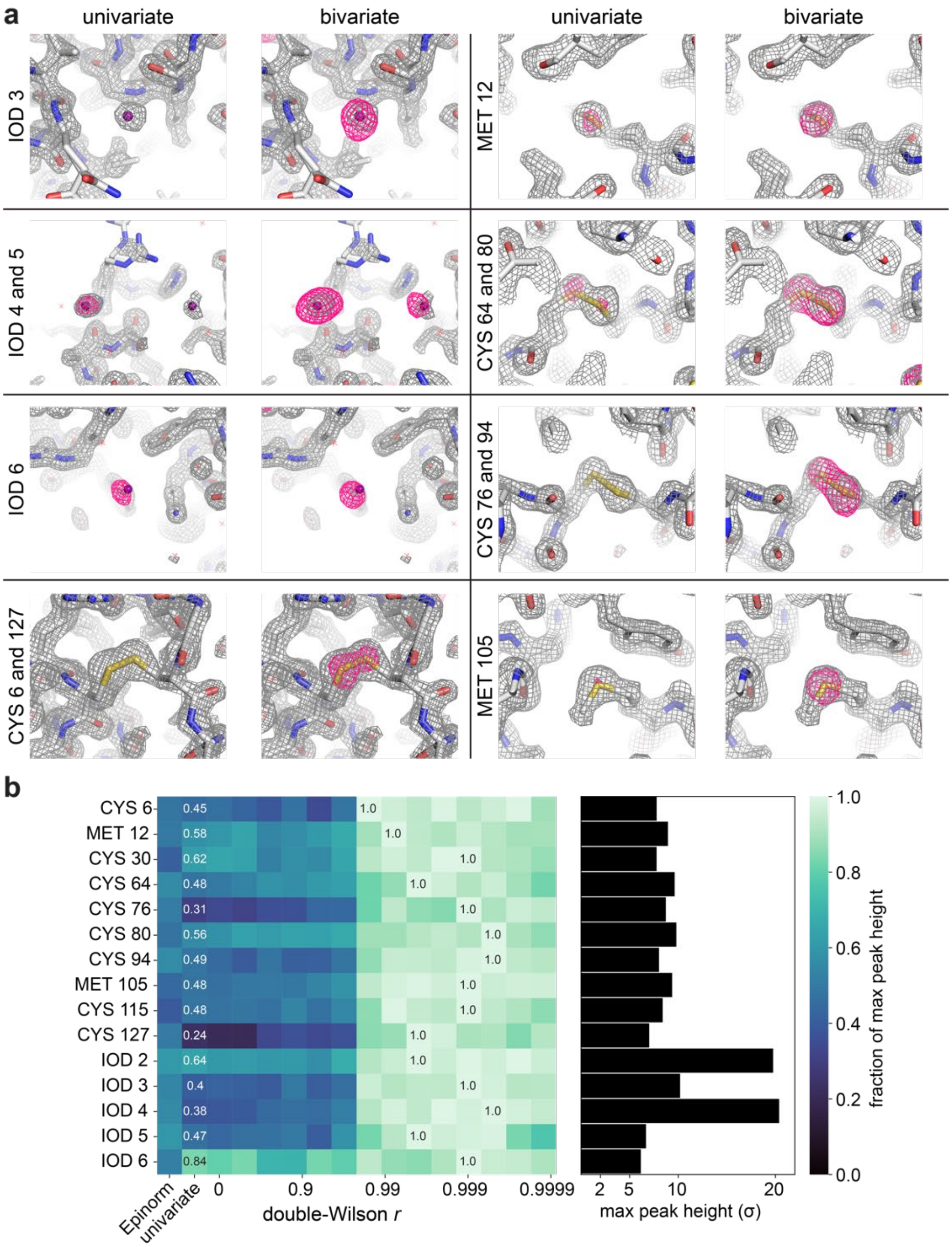

Figure S6. NaI-soaked lysozyme anomalous omit peaks improve after scaling with a bivariate prior. a) Comparisons between anomalous omit peaks merged with a bivariate prior and merged with a univariate prior. The observed electron density map ($2mF_o$-DF_c) in gray is contoured at 1.5σ and the anomalous difference omit map in magenta is contoured at 4σ. **b)** Peak heights of the anomalous difference peaks across *r*. **Left:** heatmap showing the fraction of the tallest peak across *r* for each anomalous scatterer in lysozyme. **Right:** absolute peak height of the tallest peak for each anomalous scatterer in lysozyme.

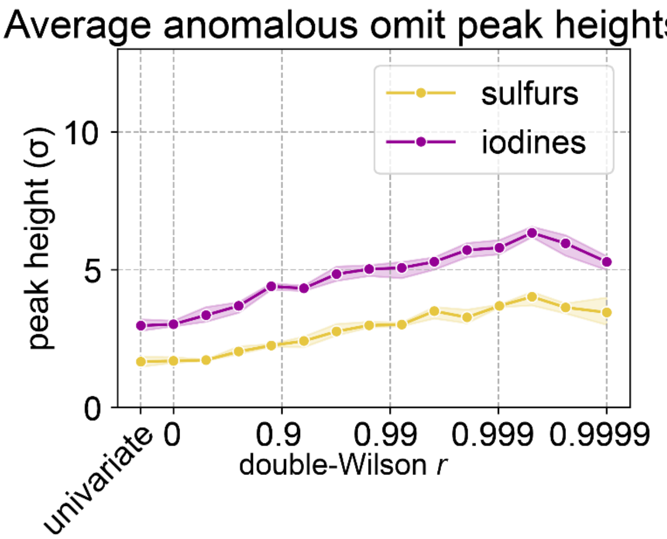

Figure S7. NaI-soaked lysozyme anomalous omit peaks on a small fraction of the data after scaling with a bivariate prior. Dependence of average iodine and sulfur anomalous peak height on *r*, after scaling with the first eighth of the dataset images. Additional peak heights are plotted for merging with the univariate prior. Shaded band represents 95% confidence interval over three independent scaling repeats.

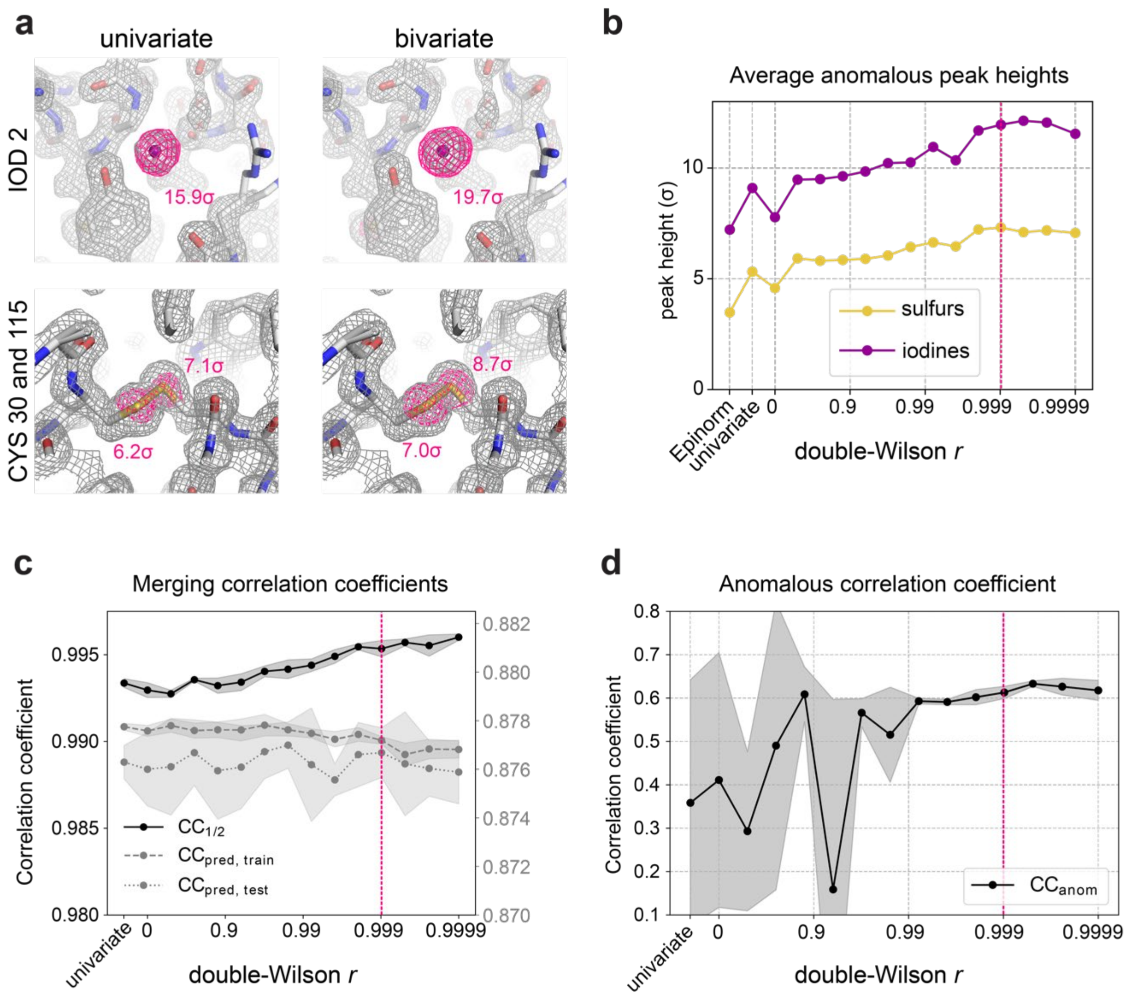

Figure S8. Anomalous signal in a Laue diffraction experiment with Laue-DIALS and a bivariate prior. a) Comparisons between anomalous omit peaks after scaling and merging with univariate or bivariate priors ($r = 0.999$ for the bivariate prior, marked in panels **b-d** with a dashed magenta line). The observed electron density map ($2mF_o$-DF_c) in gray is contoured at 1.5σ and the anomalous difference omit map in magenta is contoured at 6σ for IOD 2 and 4σ for CYS 30 and 115. **b)** Dependence of average iodine and sulfur anomalous peak height on r. Additional peak heights are plotted for merging with the univariate prior or with Epinorm. **c)** Merging correlation coefficients of the lysozyme dataset across double-Wilson r values. The y-axis labels for the $CC_{1/2}$ are on the left, and the y-axis labels for the CC_{pred} are on the right. A test set of 10% of observations were held out during scaling and merging to evaluate performance of the scaling model, yielding $CC_{\text{pred, test}}$ for the test set, and $CC_{\text{pred, train}}$ for the 90% of data used during scaling. The shaded confidence interval of the $CC_{1/2}$ curve represents the standard deviation over three half-dataset repeats. **d)** Anomalous correlation coefficient, CC_{pred}, of the lysozyme dataset across r. The shaded confidence interval represents the standard deviation over three random half-dataset partitions.

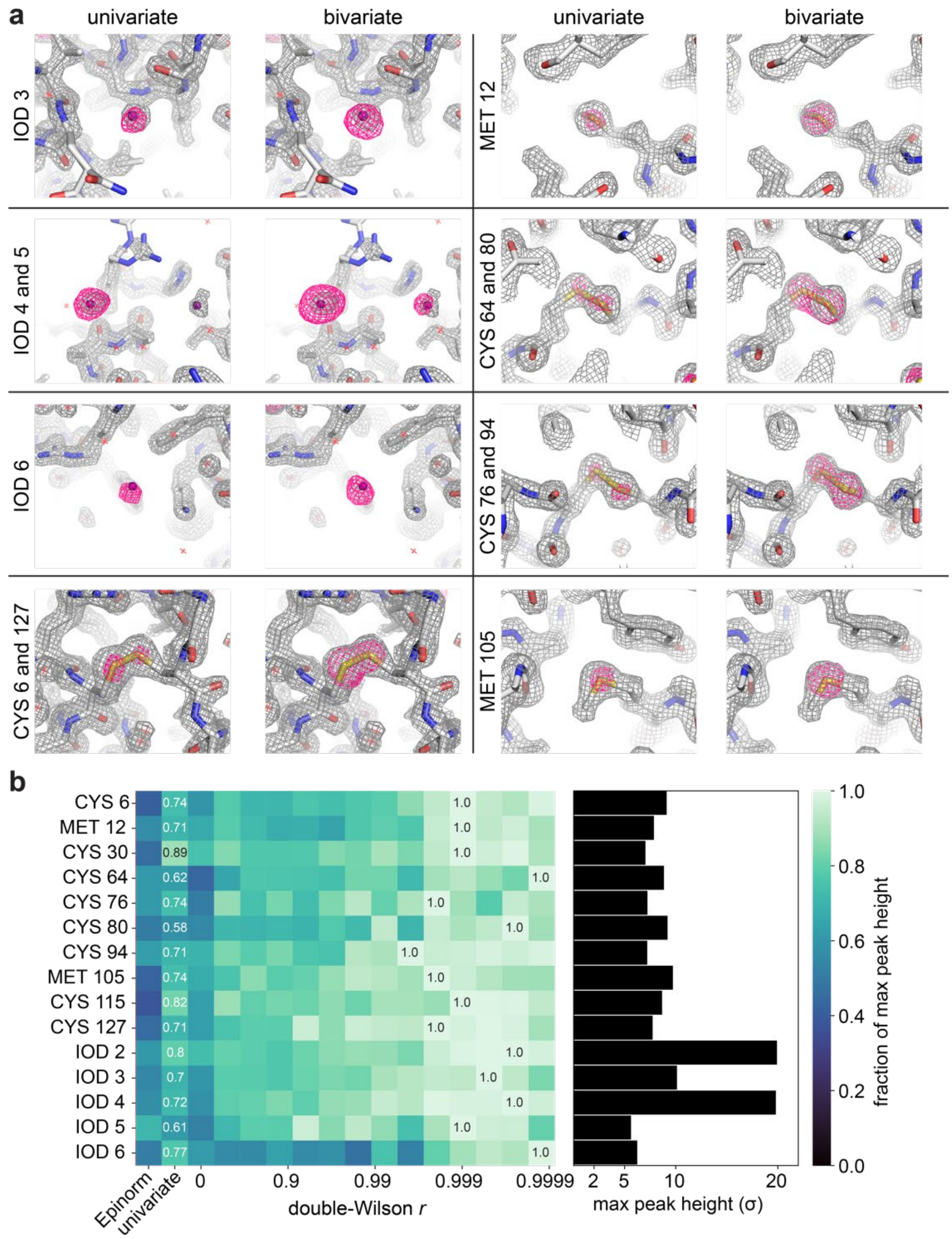

Figure S9. NaI-soaked lysozyme anomalous omit peaks integrated in Laue-DIALS, after scaling with a bivariate prior. a) Comparisons between anomalous omit peaks merged with a bivariate prior and merged with a univariate prior. The observed electron density map ($2mF_o$-DF_c) in gray is contoured at 1.5σ and the anomalous difference omit map in magenta is contoured at 4σ. **b)** Peak heights of the anomalous difference peaks across *r*. **Left:** heatmap showing the fraction of the tallest peak across *r* for each anomalous scatterer in lysozyme. **Right:** absolute peak height of the tallest peak for each anomalous scatterer in lysozyme.

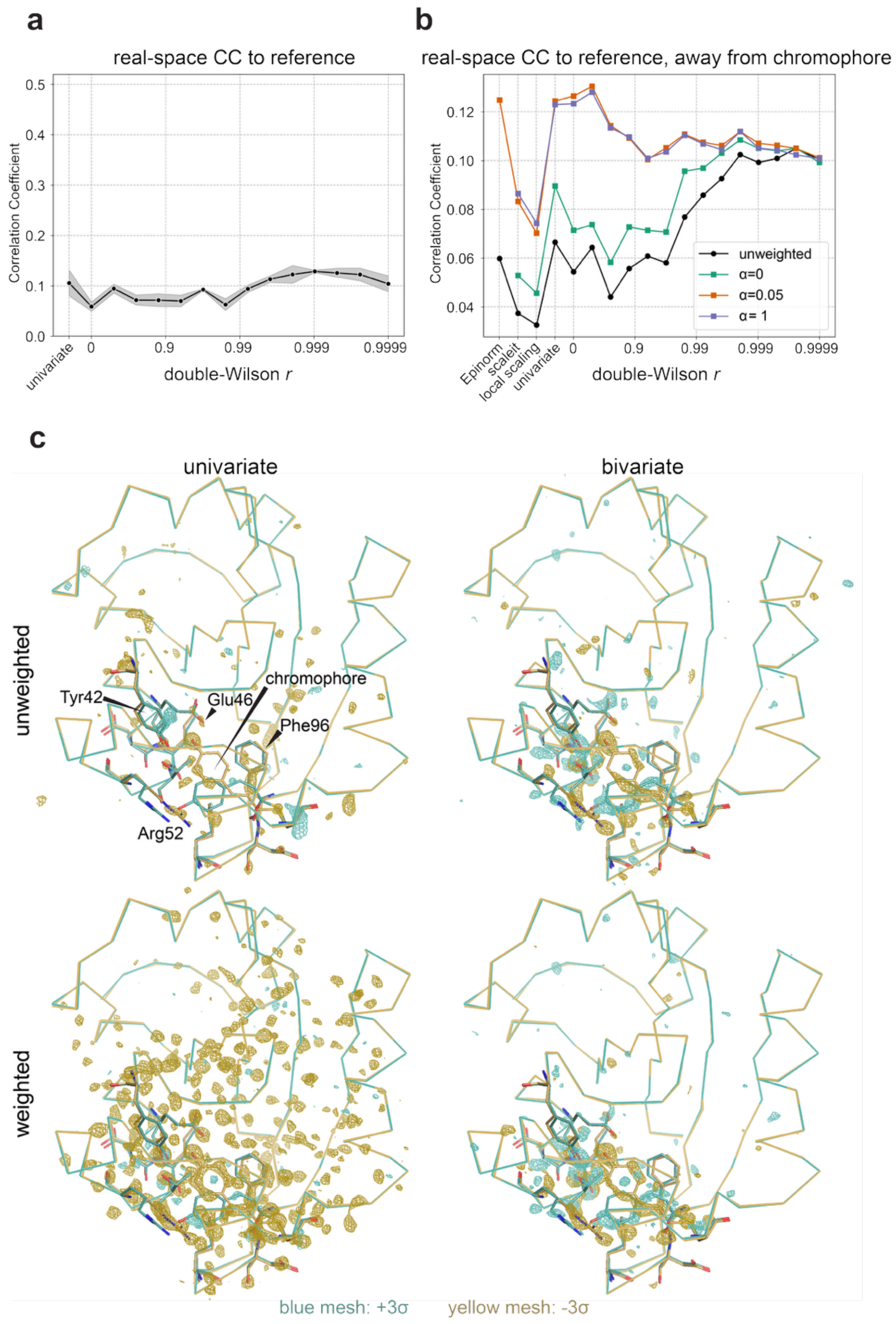

Figure S10. PYP difference maps. a) Real-space correlation coefficient between the observed and expected difference maps, in the region within 10 Å of the chromophore, for a random eighth of the data. Shaded band represents 95% confidence interval over three independent scaling repeats. **b)** Real-space correlation coefficient between the observed and expected difference maps in the region more than 10 Å away from the chromophore. **c)** Weighted and unweighted difference maps scaled with a univariate and bivariate prior, r=0.99976. Difference maps are contoured to ±3σ and are colored as indicated. The teal and yellow models are the 2ms and off structures, respectively. The region within 10 Å of the chromophore is shown as sticks.

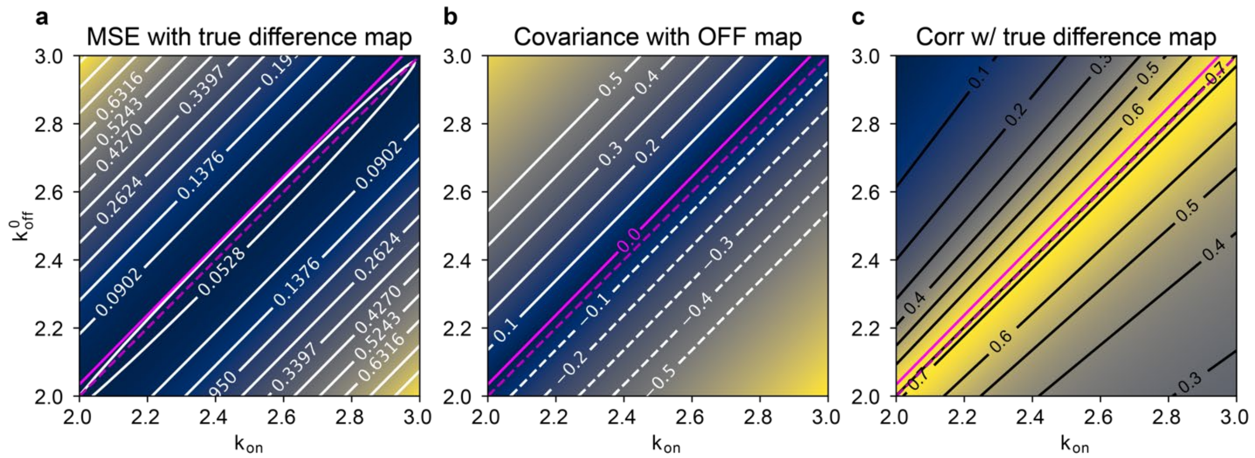

Figure S11. Negative correlation with the unperturbed state electron density can be removed without degrading difference map quality. a) the mean squared error (MSE) between a scaled difference map $(kF^{on} - k'F^{off})\exp(i\phi^{off})$ and the true, simulated difference map between ground state and excited state ($\boldsymbol{F}^{es} - \boldsymbol{F}^{gs}$) plotted across k and k'.**b)** The covariance between the scaled difference map and the unperturbed (OFF) electron density map**. c**) The Pearson correlation between the scaled difference map and the true difference map. Magenta solid lines: the line of 0 covariance between the scaled difference map and the unperturbed map $\boldsymbol{F}^{off}$. Magenta dashed lines: $k = k'$. See **Supporting Information** for notation. White lines: isocontour lines for each plot with contour isovalues shown (solid contour lines for positive values, dashed contour lines for negative values).

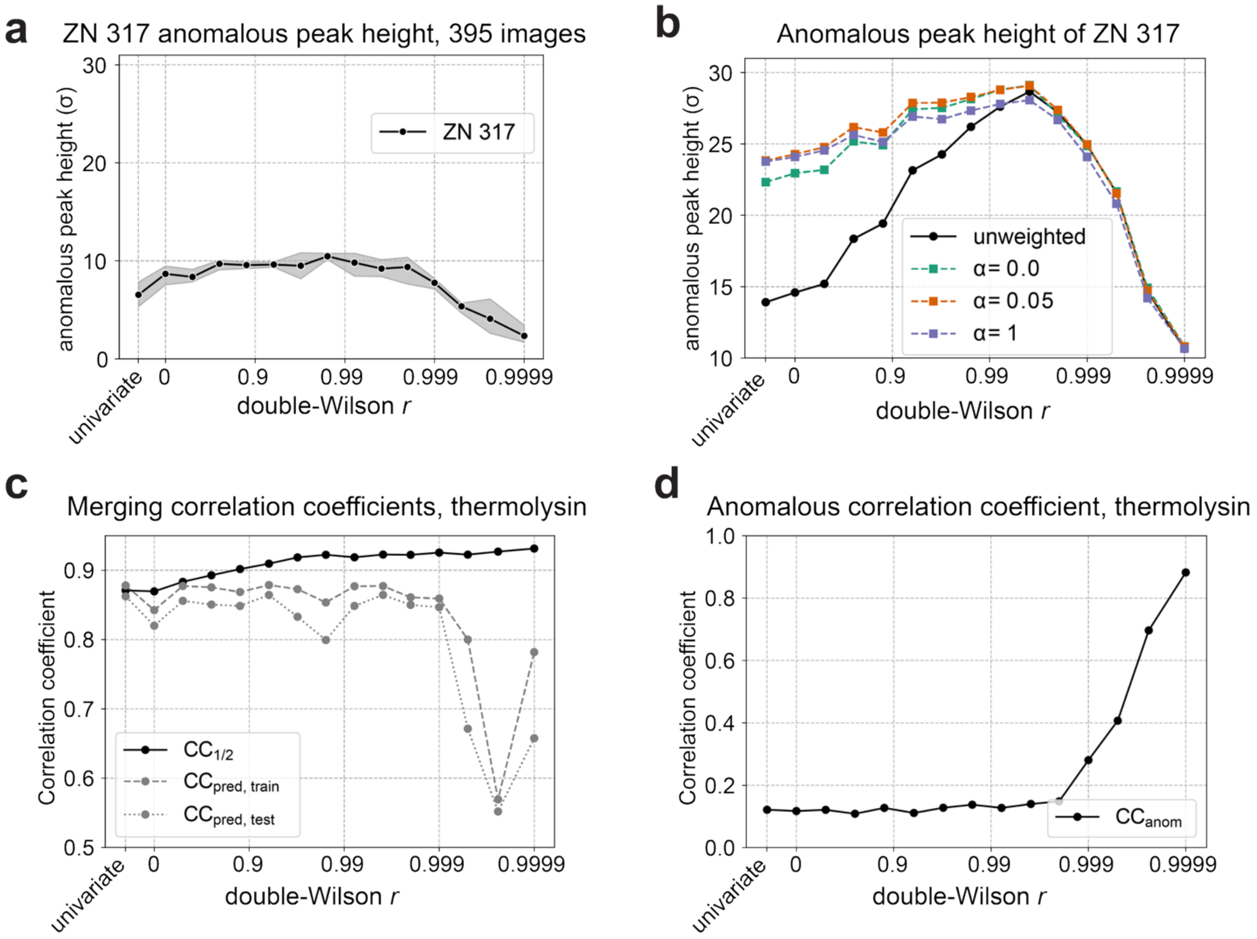

Figure S12. Anomalous differences in thermolysin omit maps. a) Dependence of average ZN317 anomalous peak height on *r*, after scaling with the first eighth of the dataset images (395 of 3160 images). Shaded band represents 95% confidence interval over three independent scaling repeats. **b)** The anomalous peak height of ZN 317 of thermolysin across double-Wilson *r*, for weighted (α=0, green; α=0.05, red; α=1, blue) and unweighted (black) anomalous differences. Shaded band represents 95% confidence interval over three independent scaling repeats. **c)** The $CC_{1/2}$, $CC_{pred,\ train}$, and $CC_{pred,\ test}$ merging statistics for the thermolysin dataset across *r*. **d)** Anomalous correlation coefficient for the thermolysin dataset across *r*. We note that the anomalous correlation coefficient, which is sensitive to systematic errors, artifactually increases past *r*=0.999.

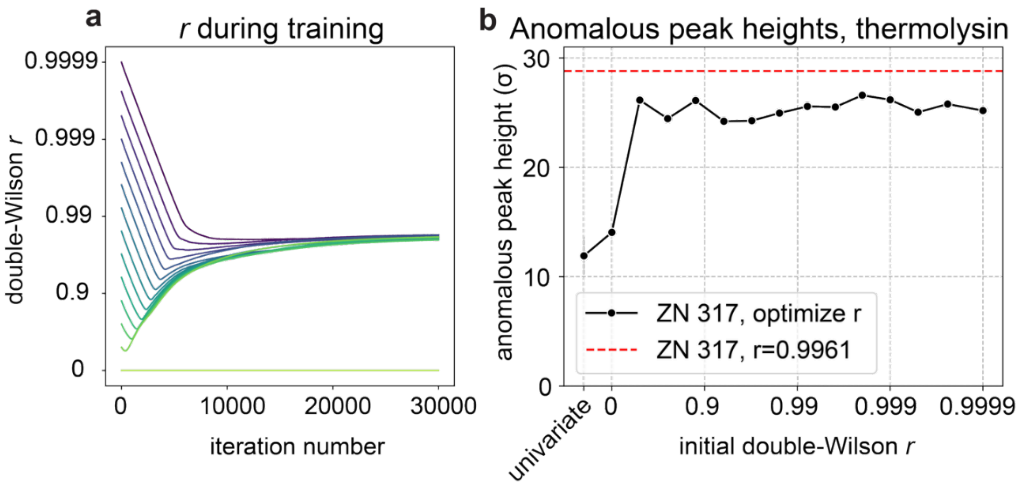

Figure S13. Direct optimization of the double-Wilson r converges on a near-optimal peak height. a) double-Wilson *r* during optimization across fourteen runs, each starting from a different *r* value. **b)** Anomalous peak height of ZN 317 after directly optimizing *r*, for many initial values of *r* (black line), along with the best *r* determined by hyperparameter sweep (dashed red line).

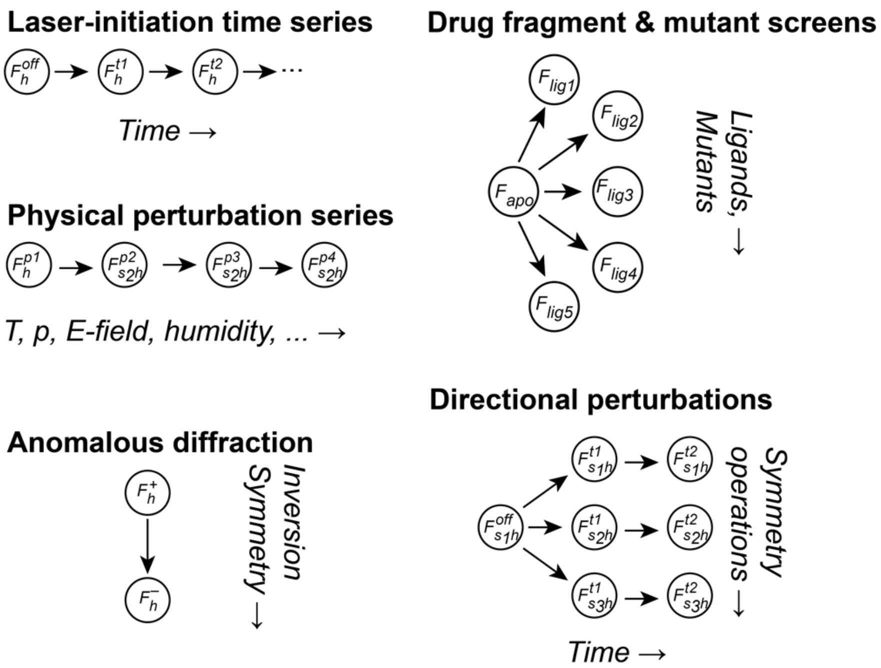

Figure S14. Modeling correlations of structure factors in comparative crystallography experiments. Correlations in many experiments are well approximated by acyclic graphical models for which the joint prior distribution of structure factor amplitudes F_h can be calculated analytically. $t_1, t_2, \ldots$ represent timepoints; $s_1, s_2, \ldots$ represent symmetry operations; $p_1, p_2, \ldots$ represent perturbations due to, e.g., temperature T, pressure p, or electric field; F^+ and F^- are reflections related by Friedel's law.

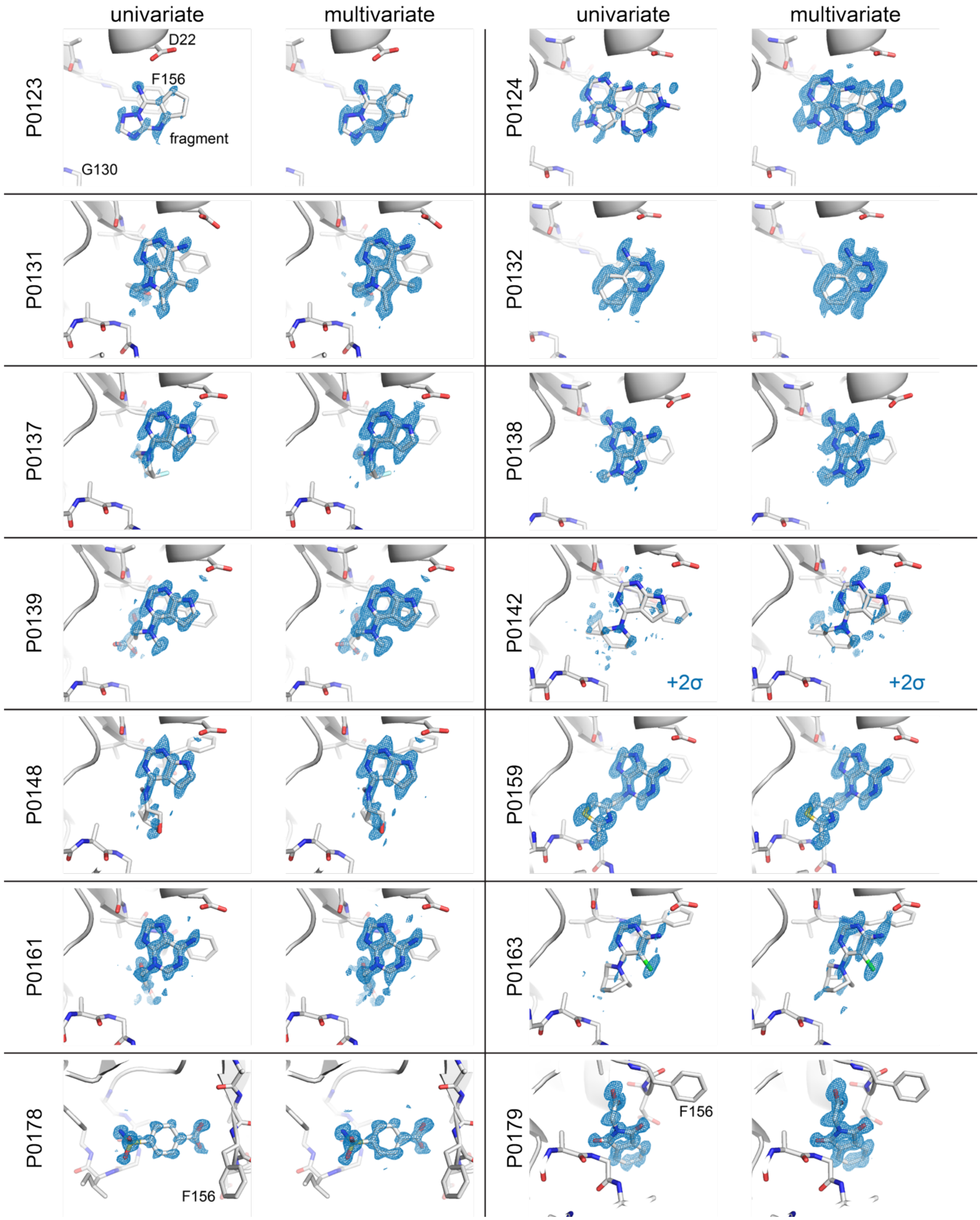

Figure S15. **Comparison of fragment screening difference maps scaled with a univariate and multivariate prior.** F_{holo} - F_{apo} difference maps from fragment screening of Mac1, scaled with a univariate and multivariate prior. All maps carved to 1.5 Å of the ligand and contoured to +3σ unless indicated.

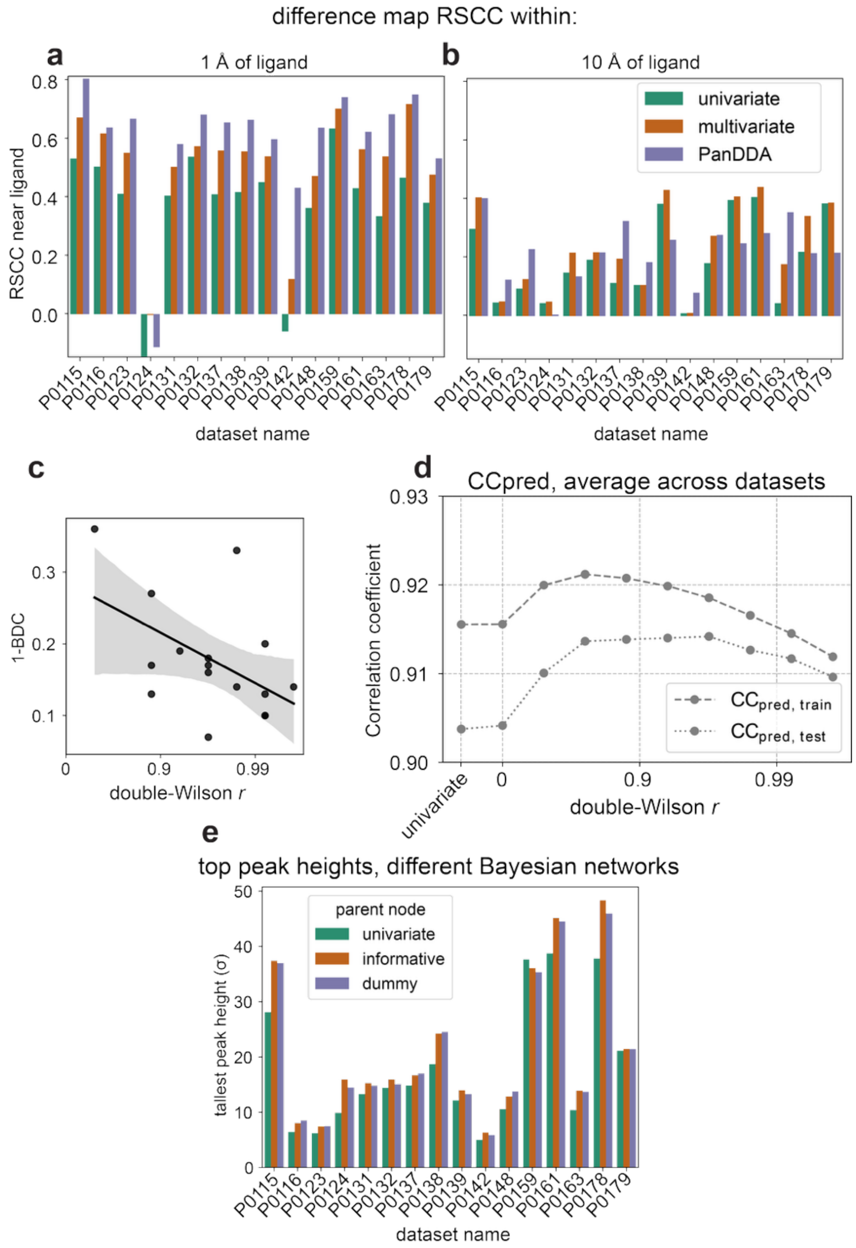

Figure S16. Fragment screening dataset statistics.

Bar plots of the real-space correlation coefficient between difference maps obtained by scaling with a univariate (green) or multivariate (orange) prior and F_c-F_c difference map generated from PanDDA models, compared to the real-space correlation coefficient between PanDDA z-maps and F_c-F_c difference maps (purple). This is repeated for a mask within **a)** 1 Å and **b)** 10 Å of the ligand. **c)** Plot of the optimal double-Wilson *r* value for each dataset, against the PanDDA 1 minus background data correction parameter, a proxy for the combined effects of occupancy and crystal idiosyncracies (see **Results**). The x-axis is on a log scale and the Pearson $r = 0.505$, with $p < 0.05$. Shaded band represents the 95% confidence interval for the trend line. **d)** Average CC_{pred} across the *holo* and *apo* datasets for each value of the double-Wilson *r*. **e)** Sum of top 3 ligand peaks for each difference map after merging with a univariate prior, with an informative parent node (**Figure S13**), or a dummy parent node—a copy of the *apo* dataset with errors in observed intensities inflated ~1414 times.

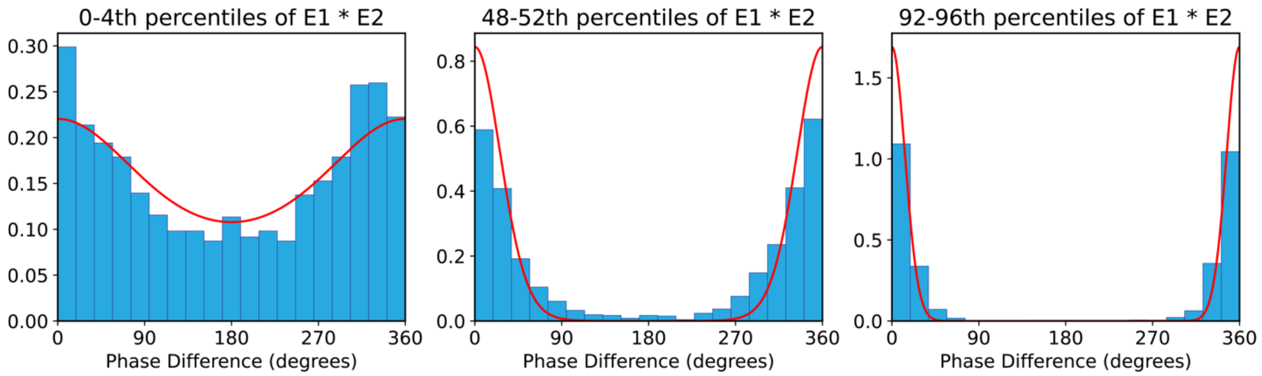

Figure S17. Von Mises statistics of phase differences. Phase differences for acentric reflections (blue bars) calculated from structure factors of PTP-1B in the absence and presence of the TCS-401 inhibitor. Von Mises distribution (red line) fit to the phase differences.

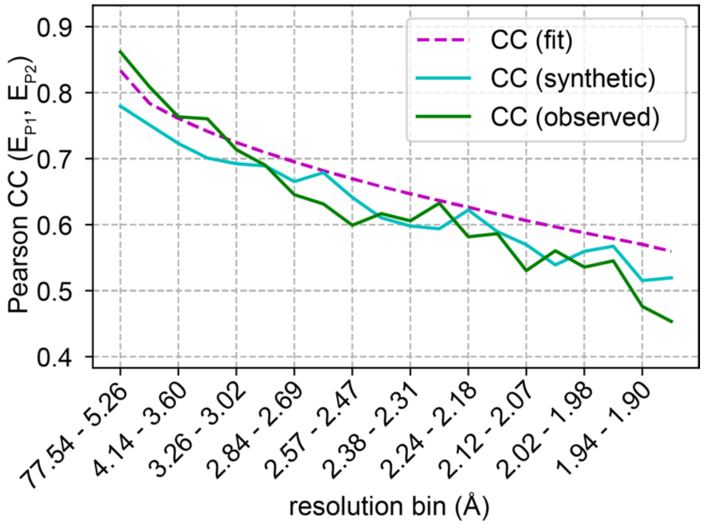

Figure S18. Resolution dependence of the correlation coefficient (CC) between related datasets. Green line: resolution-binned correlation coefficients between normalized structure factor amplitudes of PTP-1B apo and bound to the TCS-401 inhibitor. Magenta line: fit CC, $a = 0.91$ and $b = 0.71$ describing the inferred correlation between the true structure factor amplitudes (**Supplementary Information, "The bivariate Wilson distribution").** Cyan line: CC between two synthetic datasets with correlations described by the same parameters, combined with structure factor amplitudes and measurement errors drawn from their observed values.

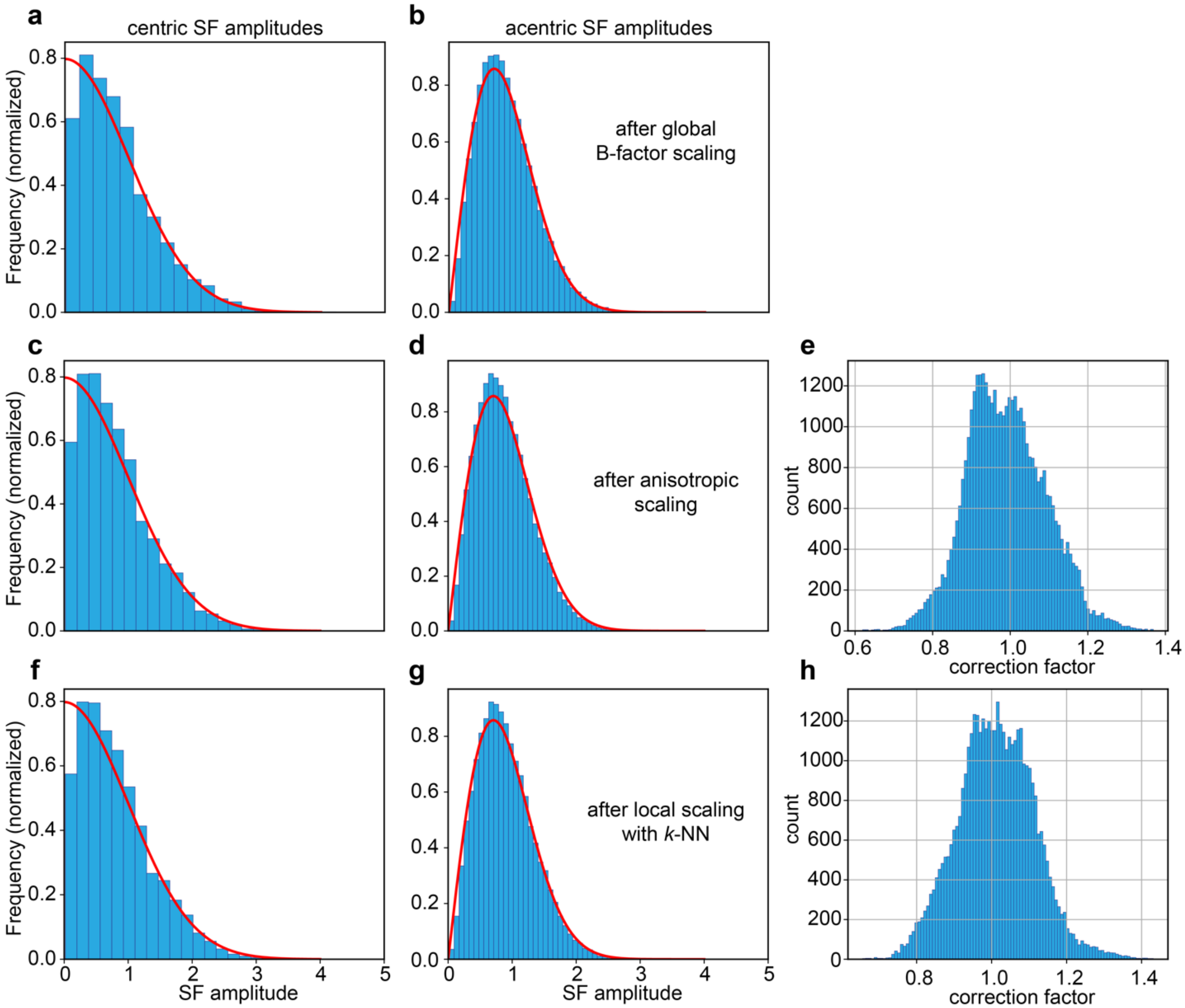

Figure S19. Normalization and Wilson statistics of structure factors. a-b) Distribution of structure factor amplitudes after global B-factor scaling for centric (**a**) and acentric (**b**) structure factor amplitudes. Red curves: expected distribution of normalized structure factor amplitudes under the Wilson distribution. **c-d)** Distribution of structure factor amplitudes after simple anisotropic scaling for centric (**c**) and acentric (**d**) structure factor amplitudes. Red curves again represent the Wilson distribution. **e)** Distribution of correction factors for a Fourier series correction of structure factor amplitudes (see **Supplementary Information**). **f-g)** Distribution of normalized structure factor amplitudes after additional correction based on *k*-nearest neighbor ridge regression for centric (**f**) and acentric (**g**) reflections. Red curves again represent the Wilson distribution. Data for apo PTP-1B (*60*). **h).** Distribution of correction factors relative to naïve anisotropic scaling.

REFERENCES AND NOTES

1. J. R. Kiefer, C. Mao, J. C. Braman, L. S. Beese, Visualizing DNA replication in a catalytically active Bacillus DNA polymerase crystal. *Nature* **391**, 304–307 (1998).

2. N. Chim, R. A. Meza, A. M. Trinh, K. Yang, J. C. Chaput, Following replicative DNA synthesis by time-resolved x-ray crystallography. *Nat. Commun.* **12**, 2641 (2021).

3. T. Nakamura, Y. Zhao, Y. Yamagata, Y.-j. Hua, W. Yang, Watching DNA polymerase η make a phosphodiester bond. *Nature* **487**, 196–201 (2012).

4. P. Mehrabi, E. C. Schulz, R. Dsouza, H. M. Müller-Werkmeister, F. Tellkamp, R. J. D. Miller, E. F. Pai, Time-resolved crystallography reveals allosteric communication aligned with molecular breathing. *Science* **365**, 1167–1170 (2019).

5. T. Gruhl, T. Weinert, M. J. Rodrigues, C. J. Milne, G. Ortolani, K. Nass, E. Nango, S. Sen, P. J. M. Johnson, C. Cirelli, A. Furrer, S. Mous, P. Skopintsev, D. James, F. Dworkowski, P. Båth, D. Kekilli, D. Ozerov, R. Tanaka, H. Glover, C. Bacellar, S. Brünle, C. M. Casadei, A. D. Diethelm, D. Gashi, G. Gotthard, R. Guixà-González, Y. Joti, V. Kabanova, G. Knopp, E. Lesca, P. Ma, I. Martiel, J. Mühle, S. Owada, F. Pamula, D. Sarabi, O. Tejero, C. J. Tsai, N. Varma, A. Wach, S. Boutet, K. Tono, P. Nogly, X. Deupi, S. Iwata, R. Neutze, J. Standfuss, G. Schertler, V. Panneels, Ultrafast structural changes direct the first molecular events of vision. *Nature* **615**, 939–944 (2023).

6. T. R. M. Barends, L. Foucar, A. Ardevol, K. Nass, A. Aquila, S. Botha, R. B. Doak, K. Falahati, E. Hartmann, M. Hilpert, M. Heinz, M. C. Hoffmann, J. Köfinger, J. E. Koglin, G. Kovacsova, M. Liang, D. Milathianaki, H. T. Lemke, J. Reinstein, C. M. Roome, R. L. Shoeman, G. J. Williams, I. Burghardt, G. Hummer, S. Boutet, I. Schlichting, Direct observation of ultrafast collective motions in CO myoglobin upon ligand dissociation. *Science* **350**, 445–450 (2015).

7. T. R. M. Barends, A. Gorel, S. Bhattacharyya, G. Schirò, C. Bacellar, C. Cirelli, J. P. Colletier, L. Foucar, M. L. Grünbein, E. Hartmann, M. Hilpert, J. M. Holton, P. J. M. Johnson, M. Kloos, G. Knopp, B. Marekha, K. Nass, G. Nass Kovacs, D. Ozerov, M. Stricker, M. Weik, R. B. Doak, R. L. Shoeman, C. J. Milne, M. Huix-Rotllant, M. Cammarata, I. Schlichting, Influence

of pump laser fluence on ultrafast myoglobin structural dynamics. *Nature* **626**, 905–911 (2024).

8. V. Šrajer, Z. Ren, T. Y. Teng, M. Schmidt, T. Ursby, D. Bourgeois, C. Pradervand, W. Schildkamp, M. Wulff, K. Moffat, Protein conformational relaxation and ligand migration in myoglobin: A nanosecond to millisecond molecular movie from time-resolved Laue x-ray diffraction. *Biochemistry* **40**, 13802–13815 (2001).

9. S. Mous, G. Gotthard, D. Ehrenberg, S. Sen, T. Weinert, P. J. M. Johnson, D. James, K. Nass, A. Furrer, D. Kekilli, P. Ma, S. Brünle, C. M. Casadei, I. Martiel, F. Dworkowski, D. Gashi, P. Skopintsev, M. Wranik, G. Knopp, E. Panepucci, V. Panneels, C. Cirelli, D. Ozerov, G. F. X. Schertler, M. Wang, C. Milne, J. Standfuss, I. Schapiro, J. Heberle, P. Nogly, Dynamics and mechanism of a light-driven chloride pump. *Science* **375**, 845–851 (2022).

10. J. H. Yun, X. Li, J. Yue, J. H. Park, Z. Jin, C. Li, H. Hu, Y. Shi, S. Pandey, S. Carbajo, S. Boutet, M. S. Hunter, M. Liang, R. G. Sierra, T. J. Lane, L. Zhou, U. Weierstall, N. A. Zatsepin, M. Ohki, J. R. H. Tame, S. Y. Park, J. C. H. Spence, W. Zhang, M. Schmidt, W. Lee, H. Liu, Early-stage dynamics of chloride ion-pumping rhodopsin revealed by a femtosecond x-ray laser. *Proc. Natl. Acad. Sci. U.S.A.* **118**, e2020486118 (2021).

11. B. R. Lee, K. I. White, M. Socolich, M. A. Klureza, R. Henning, V. Srajer, R. Ranganathan, D. R. Hekstra, Direct visualization of electric-field-stimulated ion conduction in a potassium channel. *Cell* **188**, 77–88.e15 (2025).

12. N. E. Christou, V. Apostolopoulou, D. V. M. Melo, M. Ruppert, A. Fadini, A. Henkel, J. Sprenger, D. Oberthuer, S. Günther, A. Pateras, A. Rahmani Mashhour, O. M. Yefanov, M. Galchenkova, P. Y. A. Reinke, V. Kremling, T. E. S. Scheer, E. R. Lange, P. Middendorf, R. Schubert, E. de Zitter, K. Lumbao-Conradson, J. Herrmann, S. Rahighi, A. Kunavar, E. V. Beale, J. H. Beale, C. Cirelli, P. J. M. Johnson, F. Dworkowski, D. Ozerov, Q. Bertrand, M. Wranik, C. Bacellar, S. Bajt, S. Wakatsuki, J. A. Sellberg, N. Huse, D. Turk, H. N. Chapman, T. J. Lane, Time-resolved crystallography captures light-driven DNA repair. *Science* **382**, 1015–1020 (2023).

13. S. Günther, P. Y. A. Reinke, Y. Fernández-García, J. Lieske, T. J. Lane, H. M. Ginn, F. H. M. Koua, C. Ehrt, W. Ewert, D. Oberthuer, O. Yefanov, S. Meier, K. Lorenzen, B. Krichel, J. D. Kopicki, L. Gelisio, W. Brehm, I. Dunkel, B. Seychell, H. Gieseler, B. Norton-Baker, B. Escudero-Pérez, M. Domaracky, S. Saouane, A. Tolstikova, T. A. White, A. Hänle, M. Groessler, H. Fleckenstein, F. Trost, M. Galchenkova, Y. Gevorkov, C. Li, S. Awel, A. Peck, M. Barthelmess, F. Schlünzen, P. Lourdu Xavier, N. Werner, H. Andaleeb, N. Ullah, S. Falke, V. Srinivasan, B. A. França, M. Schwinzer, H. Brognaro, C. Rogers, D. Melo, J. J. Zaitseva-Doyle, J. Knoska, G. E. Peña-Murillo, A. R. Mashhour, V. Hennicke, P. Fischer, J. Hakanpää, J. Meyer, P. Gribbon, B. Ellinger, M. Kuzikov, M. Wolf, A. R. Beccari, G. Bourenkov, D. von Stetten, G. Pompidor, I. Bento, S. Panneerselvam, I. Karpics, T. R. Schneider, M. M. Garcia-Alai, S. Niebling, C. Günther, C. Schmidt, R. Schubert, H. Han, J. Boger, D. C. F. Monteiro, L. Zhang, X. Sun, J. Pletzer-Zelgert, J. Wollenhaupt, C. G. Feiler, M. S. Weiss, E. C. Schulz, P. Mehrabi, K. Karničar, A. Usenik, J. Loboda, H. Tidow, A. Chari, R. Hilgenfeld, C. Uetrecht, R. Cox, A. Zaliani, T. Beck, M. Rarey, S. Günther, D. Turk, W. Hinrichs, H. N. Chapman, A. R. Pearson, C. Betzel, A. Meents, X-ray screening identifies active site and allosteric inhibitors of SARS-CoV-2 main protease. *Science* **372**, 642–646 (2021).

14. M. Schuller, G. J. Correy, S. Gahbauer, D. Fearon, T. Wu, R. E. Díaz, I. D. Young, L. Carvalho Martins, D. H. Smith, U. Schulze-Gahmen, T. W. Owens, I. Deshpande, G. E. Merz, A. C. Thwin, J. T. Biel, J. K. Peters, M. Moritz, N. Herrera, H. T. Kratochvil, QCRG Structural Biology Consortium, A. Aimon, J. M. Bennett, J. Brandao Neto, A. E. Cohen, A. Dias, A. Douangamath, L. Dunnett, O. Fedorov, M. P. Ferla, M. R. Fuchs, T. J. Gorrie-Stone, J. M. Holton, M. G. Johnson, T. Krojer, G. Meigs, A. J. Powell, J. G. M. Rack, V. L. Rangel, S. Russi, R. E. Skyner, C. A. Smith, A. S. Soares, J. L. Wierman, K. Zhu, P. O'Brien, N. Jura, A. Ashworth, J. J. Irwin, M. C. Thompson, J. E. Gestwicki, F. von Delft, B. K. Shoichet, J. S. Fraser, I. Ahel, Fragment binding to the Nsp3 macrodomain of SARS-CoV-2 identified through crystallographic screening and computational docking. *Sci. Adv.* **7**, eabf8711 (2021).

15. J. A. Newman, A. Douangamath, S. Yadzani, Y. Yosaatmadja, A. Aimon, J. Brandão-Neto, L. Dunnett, T. Gorrie-stone, R. Skyner, D. Fearon, M. Schapira, F. von Delft, O. Gileadi, Structure, mechanism and crystallographic fragment screening of the SARS-CoV-2 NSP13 helicase. *Nat. Commun.* **12**, 4848 (2021).

16. D. A. Keedy, Z. B. Hill, J. T. Biel, E. Kang, T. J. Rettenmaier, J. Brandão-Neto, N. M. Pearce, F. von Delft, J. A. Wells, J. S. Fraser, An expanded allosteric network in PTP1B by multitemperature crystallography, fragment screening, and covalent tethering. *eLife* **7**, e36307 (2018).

17. A. Creon, T. Emilie S. Scheer, P. Reinke, A. R. Mashhour, S. Günther, S. Niebling, K. Schamoni-Kast, C. Uetrecht, A. Meents, H. N. Chapman, J. Sprenger, T. J. Lane, Statistical crystallography reveals an allosteric network in SARS-CoV-2 Mpro. bioRxiv 2025.01.28.635305 [Preprint] (2025). https://doi.org/10.1101/2025.01.28.635305.

18. S. Shin, New era of synchrotron radiation: Fourth-generation storage ring. *AAPPS Bull.* **31**, 21 (2021).

19. P. Emma, R. Akre, J. Arthur, R. Bionta, C. Bostedt, J. Bozek, A. Brachmann, P. Bucksbaum, R. Coffee, F. J. Decker, Y. Ding, D. Dowell, S. Edstrom, A. Fisher, J. Frisch, S. Gilevich, J. Hastings, G. Hays, P. Hering, Z. Huang, R. Iverson, H. Loos, M. Messerschmidt, A. Miahnahri, S. Moeller, H. D. Nuhn, G. Pile, D. Ratner, J. Rzepiela, D. Schultz, T. Smith, P. Stefan, H. Tompkins, J. Turner, J. Welch, W. White, J. Wu, G. Yocky, J. Galayda, First lasing and operation of an ångstrom-wavelength free-electron laser. *Nat. Photonics* **4**, 641–647 (2010).

20. D. Reschke, D. Hamburg, Recent Progress with Eu-XFEL. *SFR, Canada, USA* **2015**, (2015).

21. J. L. Olmos Jr., S. Pandey, J. M. Martin-Garcia, G. Calvey, A. Katz, J. Knoska, C. Kupitz, M. S. Hunter, M. Liang, D. Oberthuer, O. Yefanov, M. Wiedorn, M. Heyman, M. Holl, K. Pande, A. Barty, M. D. Miller, S. Stern, S. Roy-Chowdhury, J. Coe, N. Nagaratnam, J. Zook, J. Verburgt, T. Norwood, I. Poudyal, D. Xu, J. Koglin, M. H. Seaberg, Y. Zhao, S. Bajt, T. Grant, V. Mariani, G. Nelson, G. Subramanian, E. Bae, R. Fromme, R. Fung, P. Schwander, M. Frank, T. A. White, U. Weierstall, N. Zatsepin, J. Spence, P. Fromme, H. N. Chapman, L. Pollack, L. Tremblay, A. Ourmazd, G. N. Phillips Jr., M. Schmidt, Enzyme intermediates captured "on the fly" by mix-and-inject serial crystallography. *BMC Biol.* **16**, 59 (2018).

22. M. Schmidt, Mix and inject: Reaction initiation by diffusion for time-resolved macromolecular crystallography. *Adv. Condens. Matter Phys.* **2013**, doi.org/10.1155/2013/167276 (2013).

23. G. D. Calvey, A. M. Katz, L. Pollack, Microfluidic mixing injector holder enables routine structural enzymology measurements with mix-and-inject serial crystallography using x-ray free electron lasers. *Anal. Chem.* **91**, 7139–7144 (2019).

24. S. Pandey, G. Calvey, A. M. Katz, T. N. Malla, F. H. M. Koua, J. M. Martin-Garcia, I. Poudyal, J. H. Yang, M. Vakili, O. Yefanov, K. A. Zielinski, S. Bajt, S. Awel, K. Doerner, M. Frank, L. Gelisio, R. Jernigan, H. Kirkwood, M. Kloos, J. Koliyadu, V. Mariani, M. D. Miller, G. Mills, G. Nelson, J. L. Olmos Jr., A. Sadri, T. Sato, A. Tolstikova, W. Xu, A. Ourmazd, J. C. H. Spence, P. Schwander, A. Barty, H. N. Chapman, P. Fromme, A. P. Mancuso, G. N. Phillips Jr., R. Bean, L. Pollack, M. Schmidt, Observation of substrate diffusion and ligand binding in enzyme crystals using high-repetition-rate mix-and-inject serial crystallography. *IUCrJ* **8**, 878–895 (2021).

25. J. R. Stagno, Y. Liu, Y. R. Bhandari, C. E. Conrad, S. Panja, M. Swain, L. Fan, G. Nelson, C. Li, D. R. Wendel, T. A. White, J. D. Coe, M. O. Wiedorn, J. Knoska, D. Oberthuer, R. A. Tuckey, P. Yu, M. Dyba, S. G. Tarasov, U. Weierstall, T. D. Grant, C. D. Schwieters, J. Zhang, A. R. Ferré-D'Amaré, P. Fromme, D. E. Draper, M. Liang, M. S. Hunter, S. Boutet, K. Tan, X. Zuo, X. Ji, A. Barty, N. A. Zatsepin, H. N. Chapman, J. C. H. Spence, S. A. Woodson, Y.-X. Wang, Structures of riboswitch RNA reaction states by mix-and-inject XFEL serial crystallography. *Nature* **541**, 242–246 (2017).

26. A. Douangamath, D. Fearon, P. Gehrtz, T. Krojer, P. Lukacik, C. D. Owen, E. Resnick, C. Strain-Damerell, A. Aimon, P. Ábrányi-Balogh, J. Brandão-Neto, A. Carbery, G. Davison, A. Dias, T. D. Downes, L. Dunnett, M. Fairhead, J. D. Firth, S. P. Jones, A. Keeley, G. M. Keserü, H. F. Klein, M. P. Martin, M. E. M. Noble, P. O'Brien, A. Powell, R. N. Reddi, R. Skyner, M. Snee, M. J. Waring, C. Wild, N. London, F. von Delft, M. A. Walsh, Crystallographic and electrophilic fragment screening of the SARS-CoV-2 main protease. *Nat. Commun.* **11**, 5047 (2020).

27. T. S. Mehlman, J. T. Biel, S. M. Azeem, E. R. Nelson, S. Hossain, L. Dunnett, N. G. Paterson, A. Douangamath, R. Talon, D. Axford, H. Orins, F. von Delft, D. A. Keedy, Room-temperature crystallography reveals altered binding of small-molecule fragments to PTP1B. *eLife* **12**, e84632 (2023).

28. A. M. Wolff, E. Nango, I. D. Young, A. S. Brewster, M. Kubo, T. Nomura, M. Sugahara, S. Owada, B. A. Barad, K. Ito, A. Bhowmick, S. Carbajo, T. Hino, J. M. Holton, D. Im, L. J. O'Riordan, T. Tanaka, R. Tanaka, R. G. Sierra, F. Yumoto, K. Tono, S. Iwata, N. K. Sauter, J. S. Fraser, M. C. Thompson, Mapping protein dynamics at high spatial resolution with temperature-jump x-ray crystallography. *Nat. Chem.* **15**, 1549–1558 (2023).

29. D. R. Hekstra, K. I. White, M. A. Socolich, R. W. Henning, V. Šrajer, R. Ranganathan, Electric-field-stimulated protein mechanics. *Nature* **540**, 400–405 (2016).

30. D. A. Keedy, L. R. Kenner, M. Warkentin, R. A. Woldeyes, J. B. Hopkins, M. C. Thompson, A. S. Brewster, A. H. van Benschoten, E. L. Baxter, M. Uervirojnangkoorn, S. E. McPhillips, J. Song, R. Alonso-Mori, J. M. Holton, W. I. Weis, A. T. Brunger, S. M. Soltis, H. Lemke, A. Gonzalez, N. K. Sauter, A. E. Cohen, H. van den Bedem, R. E. Thorne, J. S. Fraser, Mapping the conformational landscape of a dynamic enzyme by multitemperature and XFEL crystallography. *eLife* **4**, e07574 (2015).

31. P. Bryant, F. Noé, Structure prediction of alternative protein conformations. *Nat. Commun.* **15**, 7328 (2024).

32. J. B. Sumner, The isolation and crystallization of the enzyme urease: Preliminary paper. *J. Biol. Chem.* **69**, 435–441 (1926).

33. P. J. Kasvinsky, N. B. Madsen, Activity of glycogen phosphorylase in the crystalline state. *J. Biol. Chem.* **251**, 6852–6859 (1976).

34. I. Schlichting, S. C. Almo, G. Rapp, K. Wilson, K. Petratos, A. Lentfer, A. Wittinghofer, W. Kabsch, E. F. Pai, G. A. Petsko, R. S. Goody, Time-resolved x-ray crystallographic study of the conformational change in Ha-Ras p21 protein on GTP hydrolysis. *Nature* **345**, 309–315 (1990).

35. A. L. Fink, D. Kar, R. Kotin, Ribonuclease structure and catalysis: Effects of crystalline enzyme, alcohol cryosolvents, low temperatures, and product inhibition. *Biochemistry* **26**, 8571–8579 (1987).

36. S. K. Whittier, A. C. Hengge, J. P. Loria, Conformational motions regulate phosphoryl transfer in related protein tyrosine phosphatases. *Science* **341**, 899–903 (2013).

37. J. F. Acheson, L. J. Bailey, T. C. Brunold, B. G. Fox, In-crystal reaction cycle of a toluene-bound diiron hydroxylase. *Nature* **544**, 191–195 (2017).

38. A. B. Law, E. J. Fuentes, A. L. Lee, Conservation of side-chain dynamics within a protein family. *J. Am. Chem. Soc.* **131**, 6322–6323 (2009).

39. F. A. Quiocho, C. H. Mcmurray, W. N. Lipscomb, Similarities between the conformation of arsanilazotyrosine 248 of carboxypeptidase A α in the crystalline state and in solution. *Proc. Natl. Acad. Sci. U.S.A.* **69**, 2850–2854 (1972).

40. D. Sorigué, K. Hadjidemetriou, S. Blangy, G. Gotthard, A. Bonvalet, N. Coquelle, P. Samire, A. Aleksandrov, L. Antonucci, A. Benachir, S. Boutet, M. Byrdin, M. Cammarata, S. Carbajo, S. Cuiné, R. B. Doak, L. Foucar, A. Gorel, M. Grünbein, E. Hartmann, R. Hienerwadel, M. Hilpert, M. Kloos, T. J. Lane, B. Légeret, P. Legrand, Y. Li-Beisson, S. L. Y. Moulin, D. Nurizzo, G. Peltier, G. Schirò, R. L. Shoeman, M. Sliwa, X. Solinas, B. Zhuang, T. R. M. Barends, J.-P. Colletier, M. Joffre, A. Royant, C. Berthomieu, M. Weik, T. Domratcheva, K. Brettel, M. H. Vos, I. Schlichting, P. Arnoux, P. Müller, F. Beisson, Mechanism and dynamics of fatty acid photodecarboxylase. *Science* **372**, eabd5687 (2021).

41. C. Kupitz, J. L. Olmos Jr., M. Holl, L. Tremblay, K. Pande, S. Pandey, D. Oberthür, M. Hunter, M. Liang, A. Aquila, J. Tenboer, G. Calvey, A. Katz, Y. Chen, M. O. Wiedorn, J. Knoska, A. Meents, V. Majriani, T. Norwood, I. Poudyal, T. Grant, M. D. Miller, W. Xu, A. Tolstikova, A. Morgan, M. Metz, J. M. Martin-Garcia, J. D. Zook, S. Roy-Chowdhury, J. Coe, N. Nagaratnam, D. Meza, R. Fromme, S. Basu, M. Frank, T. White, A. Barty, S. Bajt, O. Yefanov, H. N. Chapman, N. Zatsepin, G. Nelson, U. Weierstall, J. Spence, P. Schwander, L. Pollack, P. Fromme, A. Ourmazd, G. N. Phillips Jr., M. Schmidt, Structural enzymology using x-ray free electron lasers. *Struct. Dyn.* **4**, 044003 (2017).

42. M. Wilamowski, D. A. Sherrell, Y. Kim, A. Lavens, R. W. Henning, K. Lazarski, A. Shigemoto, M. Endres, N. Maltseva, G. Babnigg, S. C. Burdette, V. Srajer, A. Joachimiak, Time-resolved beta-lactam cleavage by L1 metallo-beta-lactamase. *Nat. Commun.* **13**, 7379 (2022).

43. K. A. Zielinski, C. Dolamore, K. M. Dalton, N. Smith, J. Termini, R. Henning, V. Srajer, D. R. Hekstra, L. Pollack, M. A. Wilson, Resolving DJ-1 glyoxalase catalysis using mix-and-inject serial crystallography at a synchrotron. bioRxiv 2024.07.19.604369 [Preprint] (2024). https://doi.org/10.1101/2024.07.19.604369.

44. P. Nogly, T. Weinert, D. James, S. Carbajo, D. Ozerov, A. Furrer, D. Gashi, V. Borin, P. Skopintsev, K. Jaeger, K. Nass, P. Båth, R. Bosman, J. Koglin, M. Seaberg, T. Lane, D. Kekilli, S. Brünle, T. Tanaka, W. Wu, C. Milne, T. White, A. Barty, U. Weierstall, V. Panneels, E. Nango, S. Iwata, M. Hunter, I. Schapiro, G. Schertler, R. Neutze, J. Standfuss, Retinal isomerization in bacteriorhodopsin captured by a femtosecond x-ray laser. *Science* **361**, eaat0094 (2018).

45. J. Kern, R. Chatterjee, I. D. Young, F. D. Fuller, L. Lassalle, M. Ibrahim, S. Gul, T. Fransson, A. S. Brewster, R. Alonso-Mori, R. Hussein, M. Zhang, L. Douthit, C. de Lichtenberg, M. H. Cheah, D. Shevela, J. Wersig, I. Seuffert, D. Sokaras, E. Pastor, C. Weninger, T. Kroll, R. G. Sierra, P. Aller, A. Butryn, A. M. Orville, M. Liang, A. Batyuk, J. E. Koglin, S. Carbajo, S. Boutet, N. W. Moriarty, J. M. Holton, H. Dobbek, P. D. Adams, U. Bergmann, N. K. Sauter, A. Zouni, J. Messinger, J. Yano, V. K. Yachandra, Structures of the intermediates of Kok's photosynthetic water oxidation clock. *Nature* **563**, 421–425 (2018).

46. A. Bhowmick, R. Hussein, I. Bogacz, P. S. Simon, M. Ibrahim, R. Chatterjee, M. D. Doyle, M. H. Cheah, T. Fransson, P. Chernev, I. S. Kim, H. Makita, M. Dasgupta, C. J. Kaminsky, M. Zhang, J. Gätcke, S. Haupt, I. I. Nangca, S. M. Keable, A. O. Aydin, K. Tono, S. Owada, L. B. Gee, F. D. Fuller, A. Batyuk, R. Alonso-Mori, J. M. Holton, D. W. Paley, N. W. Moriarty, F. Mamedov, P. D. Adams, A. S. Brewster, H. Dobbek, N. K. Sauter, U. Bergmann, A. Zouni, J. Messinger, J. Kern, J. Yano, V. K. Yachandra, Structural evidence for intermediates during O_2 formation in photosystem II. *Nature* **617**, 629–636 (2023).

47. T. Tosha, T. Nomura, T. Nishida, N. Saeki, K. Okubayashi, R. Yamagiwa, M. Sugahara, T. Nakane, K. Yamashita, K. Hirata, G. Ueno, T. Kimura, T. Hisano, K. Muramoto, H. Sawai, H. Takeda, E. Mizohata, A. Yamashita, Y. Kanematsu, Y. Takano, E. Nango, R. Tanaka, O. Nureki, O. Shoji, Y. Ikemoto, H. Murakami, S. Owada, K. Tono, M. Yabashi, M. Yamamoto, H. Ago, S. Iwata, H. Sugimoto, Y. Shiro, M. Kubo, Capturing an initial intermediate during the P450nor enzymatic reaction using time-resolved XFEL crystallography and caged-substrate. *Nat. Commun.* **8**, 1585 (2017).

48. J. Tenboer, S. Basu, N. Zatsepin, K. Pande, D. Milathianaki, M. Frank, M. Hunter, S. Boutet, G. J. Williams, J. E. Koglin, D. Oberthuer, M. Heymann, C. Kupitz, C. Conrad, J. Coe, S. Roy-Chowdhury, U. Weierstall, D. James, D. Wang, T. Grant, A. Barty, O. Yefanov, J. Scales, C. Gati, C. Seuring, V. Srajer, R. Henning, P. Schwander, R. Fromme, A. Ourmazd, K. Moffat, J. J. van Thor, J. C. H. Spence, P. Fromme, H. N. Chapman, M. Schmidt, Time-resolved serial crystallography captures high-resolution intermediates of photoactive yellow protein. *Science* **346**, 1242–1246 (2014).

49. K. Pande, C. D. M. Hutchison, G. Groenhof, A. Aquila, J. S. Robinson, J. Tenboer, S. Basu, S. Boutet, D. P. DePonte, M. Liang, T. A. White, N. A. Zatsepin, O. Yefanov, D. Morozov, D. Oberthuer, C. Gati, G. Subramanian, D. James, Y. Zhao, J. Koralek, J. Brayshaw, C. Kupitz, C. Conrad, S. Roy-Chowdhury, J. D. Coe, M. Metz, P. L. Xavier, T. D. Grant, J. E. Koglin, G. Ketawala, R. Fromme, V. Šrajer, R. Henning, J. C. H. Spence, A. Ourmazd, P. Schwander, U. Weierstall, M. Frank, P. Fromme, A. Barty, H. N. Chapman, K. Moffat, J. J. van Thor, M. Schmidt, Femtosecond structural dynamics drives the trans/cis isomerization in photoactive yellow protein. *Science* **352**, 725–729 (2016).

50. H. Ihee, S. Rajagopal, V. Šrajer, R. Pahl, S. Anderson, M. Schmidt, F. Schotte, P. A. Anfinrud, M. Wulff, K. Moffat, Visualizing reaction pathways in photoactive yellow protein from nanoseconds to seconds. *Proc. Natl. Acad. Sci. U.S.A.* **102**, 7145–7150 (2005).

51. U. K. Genick, G. E. O. Borgstahl, K. Ng, Z. Ren, C. Pradervand, P. M. Burke, V. Šrajer, T. Y. Teng, W. Schildkamp, D. E. McRee, K. Moffat, E. D. Getzoff, Structure of a protein photocycle intermediate by millisecond time-resolved crystallography. *Science* **275**, 1471–1475 (1997).

52. U. K. Genick, Structure-factor extrapolation using the scalar approximation: Theory, applications and limitations. *Acta Crystallogr. D Biol. Crystallogr.* **63**, 1029–1041 (2007).

53. E. De Zitter, N. Coquelle, P. Oeser, T. R. M. Barends, J.-P. Colletier, Xtrapol8 enables automatic elucidation of low-occupancy intermediate-states in crystallographic studies. *Commun. Biol.* **5**, 640 (2022).

54. K. M. Dalton, J. B. Greisman, D. R. Hekstra, A unifying Bayesian framework for merging x-ray diffraction data. *Nat. Commun.* **13**, 7764 (2022).

55. H. Mai, A. Peck, K. M. Dalton, L. S. de Moraes, J. E. Burch, F. Poitevin, H. M. Nelson, Assessing the applicability of Bayesian inference for merging small molecule microED data. ChemRxiv chemrxiv-2024-62bmk [Preprint] (2024). https://doi.org/10.26434/chemrxiv-2024-62bmk.

56. K. A. Zielinski, C. Dolamore, H. K. Wang, R. W. Henning, M. A. Wilson, L. Pollack, V. Srajer, D. R. Hekstra, K. M. Dalton, Scaling and merging time-resolved pink-beam diffraction with variational inference. *Struct. Dyn.* **11**, 064301 (2024).

57. A. J. C. Wilson, The probability distribution of x-ray intensities. *Acta Crystallogr.* **2**, 318–321 (1949).

58. R. J. Read, Structure-factor probabilities for related structures. *Acta Crystallogr. A* **46**, 900–912 (1990).

59. G. Bricogne, in *Methods in Enzymology* (Academic Press, 1997), vol. 276, pp. 361–423.

60. J. B. Greisman, K. M. Dalton, C. J. Sheehan, M. A. Klureza, I. Kurinov, D. R. Hekstra, Native SAD phasing at room temperature. *Acta. Crystallogr. D Struct. Biol.* **78**, 986–996 (2022).

61. H. Hauptman, On integrating the techniques of direct methods and isomorphous replacement. I. The theoretical basis. *Acta Cryst.* **38**, 289–294 (1982).

62. K. P. Murphy, *Machine Learning: A Probabilistic Perspective* (MIT Press, 2012).

63. L. A. Aldama, K. M. Dalton, D. R. Hekstra, Correcting systematic errors in diffraction data with modern scaling algorithms. *Acta Crystallogr. D Struct. Biol.* **79**, 796–805 (2023).

64. P. A. Karplus, K. Diederichs, Linking crystallographic model and data quality. *Science* **336**, 1030–1033 (2012).

65. P. Evans, Scaling and assessment of data quality. *Acta Crystallogr. D Biol. Crystallogr.* **62**, 72–82 (2006).

66. R. A. Hewitt, K. M. Dalton, D. A. Mendez, H. K. Wang, M. A. Klureza, D. E. Brookner, J. B. Greisman, D. McDonagh, V. Šrajer, N. K. Sauter, A. S. Brewster, D. R. Hekstra, Laue-DIALS: Open-source software for polychromatic x-ray diffraction data. *Struct. Dyn.* **11**, 054701 (2024).

67. T. Ursby, D. Bourgeois, Improved estimation of structure-factor difference amplitudesfrom poorly accurate data. *Acta Cryst.* **53**, 564–575 (1997).

68. J. Kern, R. Tran, R. Alonso-Mori, S. Koroidov, N. Echols, J. Hattne, M. Ibrahim, S. Gul, H. Laksmono, R. G. Sierra, R. J. Gildea, G. Han, J. Hellmich, B. Lassalle-Kaiser, R. Chatterjee, A. S. Brewster, C. A. Stan, C. Glöckner, A. Lampe, D. DiFiore, D. Milathianaki, A. R. Fry, M. M. Seibert, J. E. Koglin, E. Gallo, J. Uhlig, D. Sokaras, T. C. Weng, P. H. Zwart, D. E. Skinner, M. J. Bogan, M. Messerschmidt, P. Glatzel, G. J. Williams, S. Boutet, P. D. Adams, A. Zouni, J. Messinger, N. K. Sauter, U. Bergmann, J. Yano, V. K. Yachandra, Taking snapshots of photosynthetic water oxidation using femtosecond x-ray diffraction and spectroscopy. *Nat. Commun.* **5**, 4371 (2014).

69. A. S. Brewster, D. G. Waterman, J. M. Parkhurst, R. J. Gildea, I. D. Young, L. J. O'Riordan, J. Yano, G. Winter, G. Evans, N. K. Sauter, Improving signal strength in serial crystallography with DIALS geometry refinement. *Acta. Crystallogr. D Struct. Biol.* **74**, 877–894 (2018).

70. A. S. Brewster, D. G. Waterman, J. M. Parkhurst, R. J. Gildea, T. M. Michels-Clark, I. D. Young, H. J. Bernstein, G. Winter, G. Evans, N. K. Sauter, Processing XFEL data with cctbx. xfel and DIALS. *Comput. Crystallogr. Newsl.* **7**, 32–53 (2016).

71. M. Uervirojnangkoorn, O. B. Zeldin, A. Y. Lyubimov, J. Hattne, A. S. Brewster, N. K. Sauter, A. T. Brunger, W. I. Weis, Enabling x-ray free electron laser crystallography for challenging biological systems from a limited number of crystals. *eLife* **4**, e05421 (2015).

72. N. M. Pearce, T. Krojer, A. R. Bradley, P. Collins, R. P. Nowak, R. Talon, B. D. Marsden, S. Kelm, J. Shi, C. M. Deane, F. von Delft, A multi-crystal method for extracting obscured crystallographic states from conventionally uninterpretable electron density. *Nat. Commun.* **8**, 15123 (2017).

73. careless-examples; https://github.com/rs-station/careless-examples.

74. dw-examples; https://github.com/Hekstra-Lab/dw-examples/.

75. abismal; https://github.com/rs-station/abismal.

76. G. N. Murshudov, A. A. Vagin, E. J. Dodson, Refinement of macromolecular structures by the maximum-likelihood method. *Acta Crystallogr. D Biol. Crystallogr.* **53**, 240–255 (1997).

77. R. J. Read, New ways of looking at experimental phasing. *Acta Crystallogr. D Biol. Crystallogr.* **59**, 1891–1902 (2003).

78. M. A. Gilles, A. Singer, A molecular prior distribution for Bayesian inference based on Wilson statistics. *Comput. Methods Programs Biomed.* **221**, 106830 (2022).

79. A. Singer, Wilson statistics: Derivation, generalization and applications to electron cryomicroscopy. *Acta Crystallogr. A Found. Adv.* **77**, 472–479 (2021).

80. G. Huldt, A. Szoke, J. Hajdu, Diffraction imaging of single particles and biomolecules. *J. Struct. Biol.* **144**, 219–227 (2003).

81. H. N. Chapman, O. M. Yefanov, K. Ayyer, T. A. White, A. Barty, A. Morgan, V. Mariani, D. Oberthuer, K. Pande, Continuous diffraction of molecules and disordered molecular crystals. *J. Appl. Cryst.* **50**, 1084–1103 (2017).

82. D. M. Blei, A. Kucukelbir, J. D. McAuliffe, Variational inference: A review for statisticians. *J. Am. Stat. Assoc.* **112**, 859–877 (2017).

83. M. I. Jordan, Z. Ghahramani, T. S. Jaakkola, L. K. Saul, An introduction to variational methods for graphical models. *Mach. Learn.* **37**, 183–233 (1999).

84. D. P. Kingma, M. Welling, Auto-encoding variational bayes. arXiv:1312.6114 [stat.ML] (2013).

85. J. V. Dillon, I. Langmore, D. Tran, E. Brevdo, S. Vasudevan, D. Moore, B. Patton, A. Alemi, M. Hoffman, R. A. Saurous, TensorFlow distributions. arXiv:1711.10604 [cs.LG] (2017).

86. M. Schmidt, V. Srajer, R. Henning, H. Ihee, N. Purwar, J. Tenboer, S. Tripathi, Protein energy landscapes determined by five-dimensional crystallography. *Acta Crystallogr. D Biol. Crystallogr.* **69**, 2534–2542 (2013).

87. M. Wojdyr, GEMMI: A library for structural biology. *J. Open Source Softw.* **7**, 4200 (2022).

88. J. B. Greisman, K. M. Dalton, D. R. Hekstra, Reciprocalspaceship: A Python library for crystallographic data analysis. *J. Appl. Cryst.* **54**, 1521–1529 (2021).

89. P. D. Adams, P. V. Afonine, G. Bunkóczi, V. B. Chen, I. W. Davis, N. Echols, J. J. Headd, L. W. Hung, G. J. Kapral, R. W. Grosse-Kunstleve, A. J. McCoy, N. W. Moriarty, R. Oeffner, R. J. Read, D. C. Richardson, J. S. Richardson, T. C. Terwilliger, P. H. Zwart, PHENIX: A comprehensive Python-based system for macromolecular structure solution. *Acta Crystallogr. D Biol. Crystallogr.* **66**, 213–221 (2010).

90. G. E. O. Borgstahl, D. R. Williams, E. D. Getzoff, 1.4 A structure of photoactive yellow protein, a cytosolic photoreceptor: Unusual fold, active site, and chromophore. *Biochemistry* **34**, 6278–6287 (1995).

91. W. L. DeLano, PyMOL: An open-source molecular graphics tool. *CCP4 Newsl. Protein Crystallogr* **40**, 82–92 (2002).

92. K. Takeda, H. Miyatake, S. Y. Park, M. Kawamoto, N. Kamiya, K. Miki, Multi-wavelength anomalous diffraction method for I and Xe atoms using ultra-high-energy x-rays from SPring-8. *J. Appl. Cryst.* **37**, 925–933 (2004).

93. D. Schwarzenbach, H. D. Flack, On the definition and practical use of crystal-based azimuthal angles. *J. Appl. Cryst.* **22**, 601–605 (1989).

94. W. Kabsch, XDS. *Acta Crystallogr. D Biol. Crystallogr.* **66**, 125–132 (2010).

95. G. Winter, D. G. Waterman, J. M. Parkhurst, A. S. Brewster, R. J. Gildea, M. Gerstel, L. Fuentes-Montero, M. Vollmar, T. Michels-Clark, I. D. Young, N. K. Sauter, G. Evans, DIALS: Implementation and evaluation of a new integration package. *Acta Crystallogr. D Struct. Biol.* **74**, 85–97 (2018).

96. J. D. Hunter, Matplotlib: A 2D graphics environment. *Comput. Sci. Eng.* **9**, 90–95 (2007).

97. C. R. Harris, K. J. Millman, S. J. van der Walt, R. Gommers, P. Virtanen, D. Cournapeau, E. Wieser, J. Taylor, S. Berg, N. J. Smith, R. Kern, M. Picus, S. Hoyer, M. H. van Kerkwijk, M. Brett, A. Haldane, J. F. del Río, M. Wiebe, P. Peterson, P. Gérard-Marchant, K. Sheppard, T. Reddy, W. Weckesser, H. Abbasi, C. Gohlke, T. E. Oliphant, Array programming with NumPy. *Nature* **585**, 357–362 (2020).

98. W. McKinney, pandas: A foundational Python library for data analysis and statistics. *Python High Perform. Sci. Comput.* **14**, 1–9 (2011).

99. P. Virtanen, R. Gommers, T. E. Oliphant, M. Haberland, T. Reddy, D. Cournapeau, E. Burovski, P. Peterson, W. Weckesser, J. Bright, S. J. van der Walt, M. Brett, J. Wilson, K. J. Millman, N. Mayorov, A. R. J. Nelson, E. Jones, R. Kern, E. Larson, C. J. Carey, İ. Polat, Y. Feng, E. W. Moore, J. V. Plas, D. Laxalde, J. Perktold, R. Cimrman, I. Henriksen, E. A. Quintero, C. R. Harris, A. M. Archibald, A. H. Ribeiro, F. Pedregosa, P. van Mulbregt, SciPy 1.0 Contributors, SciPy 1.0: Fundamental algorithms for scientific computing in Python. *Nat. Methods* **17**, 261–272 (2020).

100. M. L. Waskom, Seaborn: Statistical data visualization. *J. Open Source Softw.* **6**, 3021 (2021).

101. M. Abadi, P. Barham, J. Chen, Z. Chen, A. Davis, J. Dean, M. Devin, S. Ghemawat, G. Irving, M. Isard, M. Kudlur, J. Levenberg, R. Monga, S. Moore, D. G. Murray, B. Steiner, P. Tucker, V. Vasudevan, P. Warden, M. Wicke, Y. Yu, X. Zheng, "TensorFlow: A system for large-scale machine learning," in *12th USENIX Symposium on Operating Systems Design and Implementation (OSDI 16)* (USENIX Association, 2016), pp. 265–283.

102. H. M. Berman, T. Battistuz, T. N. Bhat, W. F. Bluhm, P. E. Bourne, K. Burkhardt, Z. Feng, G. L. Gilliland, L. Iype, S. Jain, P. Fagan, J. Marvin, D. Padilla, V. Ravichandran, B. Schneider, N. Thanki, H. Weissig, J. D. Westbrook, C. Zardecki, The Protein Data Bank. *Acta Crystallogr. D Biol. Crystallogr.* **58**, 899–907 (2002).

103. B. W. Matthews, E. W. Czerwinski, Local scaling: A method to reduce systematic errors in isomorphous replacement and anomalous scattering measurements. *Acta Crystallogr. A* **31**, 480–487 (1975).

104. P. Howell, G. Smith, Identification of heavy-atom derivatives by normal probability methods. *J. Appl. Cryst.* **25**, 81–86 (1992).

105. A. Gelman, J. Hullman, C. Wlezien, G. E. Morris, Information, incentives, and goals in election forecasts. *Judgm. Decis. Mak.* **15**, 863–880 (2020).

106. S. L. Lohr, J. M. Brick, Roosevelt predicted to win: Revisiting the 1936 Literary Digest poll. *Stat. Politics Policy* **8**, 65–84 (2017).

107. C. Kennedy, M. Blumenthal, S. Clement, J. D. Clinton, C. Durand, C. Franklin, K. McGeeney, L. Miringoff, K. Olson, D. Rivers, L. Saad, G. E. Witt, C. Wlezien, An evaluation of the 2016 election polls in the United States. *Public Opin. Q.* **82**, 1–33 (2018).

108. T. C. Terwilliger, J. Berendzen, Automated MAD and MIR structure solution. *Acta Crystallogr. D Biol. Crystallogr.* **55**, 849–861 (1999).

109. H. Cao, J. Skolnick, Time-resolved x-ray crystallography capture of a slow reaction tetrahydrofolate intermediate. *Struct. Dyn.* **6**, 024701 (2019).

110. B. Rupp, *Biomolecular Crystallography: Principles, Practice, and Application to Structural Biology* (Garland Science, ed. 1, 2010), pp. 1–809; https://doi.org/10.1201/9780429258756.

111. H. A. Hauptman, The phase problem of x-ray crystallography. *Rep. Prog. Phys.* **54**, 1427–1454 (1991).

112. R. J. Read, Improved Fourier coefficients for maps using phases from partial structures with errors. *Acta Cryst.* **42**, 140–149 (1986).

113. A. B. Yoo, M. A. Jette, M. Grondona, in *Workshop on Job Scheduling Strategies for Parallel Processing* (Springer, 2003), pp. 44–60.

114. N. S. Pannu, A. J. McCoy, R. J. Read, Application of the complex multivariate normal distribution to crystallographic methods with insights into multiple isomorphous replacement phasing. *Acta Crystallogr. D Biol. Crystallogr.* **59**, 1801–1808 (2003).

115. Z. Otwinowski, W. Minor, “Denzo and scalepack,” in *International Tables for Crystallography* (International Union of Crystallography, 2006), pp. 226–235.

116. A. J. McCoy, R. W. Grosse-Kunstleve, P. D. Adams, M. D. Winn, L. C. Storoni, R. J. Read, Phaser crystallographic software. *J. Appl. Cryst.* **40**, 658–674 (2007).

117. A. N. Popov, G. P. Bourenkov, Choice of data-collection parameters based on statistic modelling. *Acta Crystallogr. D Biol. Crystallogr.* **59**, 1145–1153 (2003).

118. J. B. Greisman, K. M. Dalton, D. E. Brookner, M. A. Klureza, C. J. Sheehan, I. S. Kim, R. W. Henning, S. Russi, D. R. Hekstra, Perturbative diffraction methods resolve a conformational switch that facilitates a two-step enzymatic mechanism. *Proc. Natl. Acad. Sci. U.S.A.* **121**, e2313192121 (2024).

119. I. I. Vorontsov, P. Coppens, On the refinement of time-resolved diffraction data: Comparison of the random-distribution and cluster-formation models and analysis of the light-induced increase in the atomic displacement parameters. *J. Synchrotron Radiat.* **12**, 488–493 (2005).

120. G. Sim, The distribution of phase angles for structures containing heavy atoms. II. A modification of the normal heavy-atom method for non-centrosymmetrical structures. *Acta Crystallogr.* **12**, 813–815 (1959).

121. S. Russi, A. González, L. R. Kenner, D. A. Keedy, J. S. Fraser, H. van den Bedem, Conformational variation of proteins at room temperature is not dominated by radiation damage. *J. Synchrotron Radiat.* **24**, 73–82 (2017).

122. J. A. Arpino, P. J. Rizkallah, D. D. Jones, Crystal structure of enhanced green fluorescent protein to 1.35 Å resolution reveals alternative conformations for Glu222. *PLOS One* **7**, e47132 (2012).

123. A. Royant, M. Noirclerc-Savoye, Stabilizing role of glutamic acid 222 in the structure of Enhanced Green Fluorescent Protein. *J. Struct. Biol.* **174**, 385–390 (2011).

124. E. D. Getzoff, K. N. Gutwin, U. K. Genick, Anticipatory active-site motions and chromophore distortion prime photoreceptor PYP for light activation. *Nat. Struct. Mol. Biol.* **10**, 663–668 (2003).

125. U. K. Genick, S. M. Soltis, P. Kuhn, I. L. Canestrelli, E. D. Getzoff, Structure at 0.85 Å resolution of an early protein photocycle intermediate. *Nature* **392**, 206–209 (1998).
